# Conditioned taste aversion versus avoidance: A re-examination of the separate processes hypothesis

**DOI:** 10.1371/journal.pone.0217458

**Published:** 2019-06-19

**Authors:** Lindsey A. Schier, Kellie M. Hyde, Alan C. Spector

**Affiliations:** 1 Department of Biological Sciences, University of Southern California, Los Angeles, California, United States of America; 2 Department of Psychology, Program in Neuroscience, Florida State University, Tallahassee, Florida, United States of America; Barnard College, UNITED STATES

## Abstract

Rats not only *avoid* ingesting a substance associated with LiCl toxicosis, but they display rejection reflexes (e.g., gapes) to its taste; this latter response is thought to reflect disgust or taste *aversion*. Prior work has shown that rats also avoid consuming foods/fluids associated with other adverse gastrointestinal (GI) effects like lactose indigestion but without the concomitant change in oromotor responses (taste reactivity; TR) indicative of aversion. Because of interpretive limitations of the methods used in those studies, we revisited the taste aversion-avoidance distinction with a design that minimized non-treatment differences among groups. Effects on intake and preference (Experiments 1a, 1b, and 2), as well as consummatory (TR, Experiment 1a and 1b) and appetitive (Progressive Ratio, Experiment 2) behaviors to the taste stimulus were assessed after training. In both experiments, rats were trained to associate 0.2% saccharin (CS) with intraduodenal infusions of LiCl, Lactose, or NaCl control. Rats trained with 18% lactose, 0.3 and 1.5 mEq/kg dose of LiCl subsequently avoided the taste CS in post-training single-bottle intake tests and two-bottle choice tests. However, only those trained with 1.5 mEq/kg LiCl displayed post-conditioning increases in taste CS-elicited aversive TR (Experiment 1a and 1b). This dose of LiCl also led to reductions in breakpoint for saccharin. The fact that conditioned avoidance is not always accompanied by changes in other common appetitive and/or consummatory indices of ingestive motivation further supports a functional dissociation between these processes, and highlights the intricacies of visceral influences on taste-guided ingestive motivation.

## Introduction

The gustatory system is the ultimate sentry of the gastrointestinal (GI) tract. Stimulation of its specialized chemoreceptors in the oral cavity evokes motor outputs that promote, in the case of potentially beneficial substances (e.g., nutrients), or deter, in the case of potentially harmful substances, ingestion. Consistent with the general heuristic put forth by Craig [1], taste-guided behaviors can be further subdivided in to those belonging to the appetitive or consummatory phases of ingestion [2]. Appetitive behaviors are typically considered goal-directed motor sequences that bring the animal into contact with substances that are nutritious or otherwise advantageous and limits contact with substances that are linked to unfavorable consequences. Taste-driven consummatory behaviors, on the other hand, are engaged when the taste stimulus makes physical contact with the oral receptors and elicits stereotypic oromotor reflexes that facilitate ingestion (e.g., licking, swallowing) or rejection (e.g., gaping, dispelling the substance from the mouth), commonly referred to as taste reactivity (TR) [2–4]. While both appetitive and consummatory behaviors *appear to be inherently linked to specific taste sensory input—*e.g., “bitter” plant alkaloids are avoided and rejected in the naïve subject—such responses can also be acquired or modified through learning about the actual postingestive visceral consequences of the food or fluid [2, 5–8]

*Conditioned taste aversion* (CTA) is the most well-studied form of taste-visceral learning, whereby normally positive (or neutral) appetitive and/or consummatory responses to a taste stimulus are replaced with avoidance and rejection responses, following its association with a negative visceral consequence [8–10]. Due in part to some of its special properties {for reviews, see [11, 12]}, great empirical effort has been invested into understanding the mechanisms through which such associations change taste-guided responding. One popular hypothesis is that the negative visceral consequences actually render the taste stimulus *unpalatable*, which then effectively dissuades intake [13–16]. Another possibility is that the experience conditions a response strategy that minimizes exposure to the stimulus, without necessarily changing its palatability *per se* [13–16]. Pelchat et al [14] aptly illustrated these two separate behavioral mechanisms with the following example: After consuming shrimp for the very first time, one person suffers a bad bout of food poisoning, while another breaks out in hives. Both people will avoid consuming shrimp in the future, but only the person that experienced the food poisoning develops a keen distaste for shrimp. The other is presumably avoiding consumption to prevent another allergic reaction. Implicit in this example are the notions that (1) different visceral consequences engage distinct responses and that (2) gross outcome measures such as how much shrimp one voluntarily consumes (i.e., intake) do not allow us to distinguish among potentially separate underlying processes.

In a conceptually innovative set of experiments, Pelchat et al [14] set out to assess this in a rodent model, specifically they tested whether qualitatively distinct visceral stimuli differentially affect taste-guided behaviors. The authors trained different groups of rats to associate a taste stimulus with either a) LiCl, the classic emetic agent used for training CTA, b) GI discomfort, induced by lactose {adult rats lack the digestive enzyme lactose, making them lactose intolerant [17]} or c) exteroceptive pain produced by foot shock. Not surprisingly, all three treatments led to reductions in intake of the associated taste. To probe the behavioral mechanism, the authors took advantage of the taste reactivity (TR) test. TR refers to the stereotypic oromotor reflexes elicited by taste stimulation, which can be generally subdivided into two categories: those that are related to the act of ingestion (i.e., ingestive TR, e.g., tongue protrusions) and those that are related to the act of rejection (i.e., aversive TR, e.g., gapes). Rodents exhibit increases in ingestive TR with increasing concentrations of inherently acceptable substances, like sucrose. Likewise, they will display increases in aversive TR (with parallel reductions in ingestive TR) with increasing concentrations of inherently unacceptable substances, like quinine. Moreover, if a normally-accepted taste solution is paired with the administration of LiCl a dramatic shift from ingestive to aversive reactivity will result. Owing to these facts, TR has been viewed as a nonverbal proxy of palatability [2, 18–21]. Accordingly, any effect the various unconditioned stimuli had on the consummatory phase responses to the taste stimulus would be taken to suggest a fundamental change in the palatability of the taste stimulus. Indeed, Pelchat et al [14] found that LiCl, but not lactose or foot shock, produced the expected shift in TR. The authors interpreted these findings to mean that LiCl, the nausea-inducing emetic in humans, conditioned a true *taste aversion*, whereas the other two stimuli conditioned avoidance of the taste without aversion, providing proof of principle for a functional dichotomy in these taste-guided behaviors. Simbayi et al [22] later replicated this study and its outcomes.

Nevertheless, there are some outstanding methodological features in the design that significantly limit interpretation. First, the training conditions differed greatly between the groups, beyond the visceral stimulus treatment. That is, whereas the LiCl group was trained to associate the taste of sucrose with experimenter-administered gavage of LiCl, irrespective of how much of the solution they consumed, a common method for training CTA, the Lactose group self-administered the lactose through ingestion. This meant that the lactose served both as the taste and the visceral stimulus. Given stimulus quality and intensity are known determinants in the development and subsequent expression of learning [11], this confound makes it is difficult to determine whether the differential outcome in TR was due to qualitative difference in the process or a quantitative difference in the strength of learning. This could importantly include qualitative and quantitative differences in the taste and visceral stimuli. Not only that, but the fact that the rats in the Lactose group were consuming the stimulus meant that their ingestive behavior was a critical determinant in the “dose” of lactose they ultimately received. Such variance in the contingencies between the taste and the visceral stimuli for the lactose and LiCl groups could foster dissimilar types of learning. Through this method of stimulus presentation, Lactose rats may have learned to escape the negative postingestive effects by limiting their consumption; no such operant relationship was explicitly in place for the LiCl rats. Finally, the critical measures reflecting putative changes in palatability, namely ingestive and aversive TR, were captured during 10-min intake tests whereby TR was stimulated only if and when the rat approached and subsequently made contact with the solution in the sipper spout. This arrangement means that the amount of taste solution contacting the receptors was not equated among groups, making direct comparisons of their relative efficacies with respect to evoking consummatory responses problematic. Moreover, the occurrence of each of the three ingestive response types and six aversive response types were noted, but the frequency of each response type was not quantified. Considering other studies have found that the frequencies of these various oromotor responses tracks with unconditioned and conditioned aspects of the taste stimulus and/or physiological state [2, 18, 23, 24], it seems possible that lactose or foot shock could have produced reductions in the incidence of particular ingestive responses, for example, indicative of a palatability shift that simply went undetected with the yes/no procedure used in previous studies.

Thus, here, we re-examined the hypothesized functional dichotomy of taste aversion and taste avoidance with purer measures of consummatory and appetitive behaviors following a taste-visceral conditioning paradigm that was designed to minimize non-treatment differences. Specifically, rats were trained to associate their consumption of the same taste stimulus (0.2% saccharin) with an intraduodenal infusion of one of three LiCl doses (Experiment 1a and 1b only), Lactose, or a NaCl control solution. This training procedure equated the conditioned stimulus (CS) and unconditioned stimulus (US) presentation conditions. Traditional intake measures (single-bottle acceptance) during and after (single-bottle acceptance and two-bottle choice) taste-visceral conditioning tracked the development and expression of taste CS avoidance. Taste-based consummatory responses were measured in a taste reactivity test before and after taste-visceral conditioning in Experiment 1a and 1b. As is typical in this test, a small infusion of the taste CS was delivered directly into the oral cavity and the resultant oromotor responses were video-recorded from an unobscured vantage point for subsequent quantification. This procedure ensures that the amount and duration of taste stimulation are matched across treatment conditions and while eliminating any appetitive behavior in the delivery of the stimulus. Finally, in Experiment 2, taste-reinforced appetitive responses were measured in progressive ratio (PR) tests before and after taste-visceral conditioning. In this test, the subject performed a response (i.e., dry licks) to obtain access to a small amount of the “sweet” taste stimulus (i.e., saccharin). The response requirement to receive that same amount of saccharin progressively increased across trials within the test. The small saccharin volume obtained in each trial minimized postingestive influences on responding and, thus effectively ensured that operant responses were being reinforced by the taste properties of the stimulus. If, as we expected, lactose conditioned an avoidance of a taste CS, without concomitant changes in ingestive and/or aversive TR, it would remain possible, that such experiences would nevertheless decrease behaviors geared at obtaining a taste CS associated with lactose in the PR test. But, as will be shown, the latter did not occur.

## Materials and methods

### Subjects

Naïve male Sprague Dawley rats (Charles River) weighing 251–288 g, 319–395 g and 278–348 g at the start of the Experiments 1a (n = 32), 1b (n = 10), and 2 (n = 32), respectively, were individually housed in hanging Polycarbonate cages in a climate-controlled room on a 12 h: 12h light: dark cycle. All behavioral procedures were conducted in the light phase, with the exception of the two-bottle tests which were run across two consecutive 24-h periods in the home cage (see below). All rats had ad libitum access to deionized water (dH_2_O) and chow (Labdiet #5001, PMI Nutrition), except as noted otherwise. All experimental protocols were approved by and conducted in accordance with the *Florida State University (Experiment 1a and 2) and University of Southern California (Experiment 1b) Animal Care and Use Committees*.

### Surgery

After an overnight fast, each rat in Experiments 1 and 2 underwent surgery to implant a chronic indwelling intraduodenal (ID) catheter and each rat in Experiment 1 also received bilateral intraoral (IO) cannulae under isoflurane anesthesia (5% induction rate, ~2–2.5% maintenance rate). To install the ID catheter, each rat was laparotomized, exposing the stomach and proximal intestine, and then a Silastic catheter (inside diameter = 0.64 mm, outside diameter = 1.19 mm, Dow Corning, Midland, MI) was introduced through a puncture wound in the greater curvature of the forestomach. The catheter was advanced through the pyloric sphincter and then anchored to the intestinal wall ~4 cm distal to the pyloric sphincter with a single stay suture and piece of Marlex mesh (Davol Inc., Cranston, RI). The puncture wound in the stomach was closed around the free end of the catheter with a purse string suture and a concentric serosal tunnel. The free end was then tunneled subcutaneously to an interscapular exit site, where it was exteriorized and connected to a Luer Lok adapter, which was mounted in a harness worn by the rat at all times (Quick Connect Harness, Strategic Applications, Inc., Lake Villa, IL). For rats in Experiment 1a and 1b, bilateral IO cannulae, consisting of PE-100 tubing and blunt 19 G stainless steel needles, were implanted according to a modified Grill and Norgren protocol [2] in the same surgery. Briefly, each IO cannula was placed just anterolateral to the second maxillary molar and then were anchored to the skull with a head cap made from four set screws and dental acrylic. Antibiotic cream was applied around the head cap at the end of the surgery to help prevent infection. Postoperative antibiotic (Baytril/Enrofloxin, 2.3 mg/kg, SC) and analgesic (Carprofen, 5 mg/kg, SC) were administered immediately after surgery and once daily for three days thereafter to further aid recovery. Rats were given a limited amount (~10 g) of chow mash (~50% powdered chow: 50% dH_2_O) after surgery and then ad lib access to chow mash and/or powdered chow for at least two more days, before being gradually introduced back onto regular pelleted chow. Because some rats later demonstrated difficulty maintaining a bodyweight above 85% of their free-feeding bodyweight while on a restricted access to water regimen, all rats were provided a jar of powdered chow in addition to the pelleted chow in the home cage for the remainder of the experiments. The ID catheter was routinely flushed with 0.5 ml of dH_2_O beginning 48-hr after surgery to maintain patency. Harness bands were adjusted daily to accommodate changes in body mass. Occasionally, rats developed local infections around the head cap. The infected site was cleaned daily with a dry cotton-tipped swab and treated with antibiotic cream or antiseptic solution, and, in some cases, if the infection persisted 5 days or more, SC Enrofloxin (2.3 mg/kg) and/or Carprofen (5 mg/kg) were administered until the infection subsided. Some rats were excluded from the experiment due to surgical complications (Experiments 1a, 1b, and 2), post-operative ID catheter failure or patency issues (Experiments 1a, 1b, and 2), IO patency issues (Experiments 1a and 1b), failure to maintain adequate body weight on the water restriction regimen (Experiment 1a and 2), or equipment malfunction (Experiment 1a and 2).

### Stimuli

Saccharin sodium salt hydrate (referred to here as saccharin, 0.2% w/v), D-lactose monohydrate (referred to here as lactose, 18% w/w), sodium chloride (NaCl, 0.15 M), and lithium chloride (LiCl, 0.15 M) solutions were made with dH_2_O. The Lactose, NaCl, and LiCl solutions were prepared the afternoon prior to the training or testing sessions and then lactose was left to stir overnight. To fully dissolve lactose, the solution was stirred on low heat for approximately 5 minutes. The following morning, high and low concentrations of LiCl were made by diluting the 0.15 M LiCl stock solution to 67% and 7%, respectively, with equimolar NaCl (dosage details in *Procedures* section below). An intermediate LiCl dose was used for a follow-up experiment (Experiment 1b, see below). This was made in a similar fashion by mixing 14% 0.15 M LiCl with 86% 0.15 M NaCl. Even though these solutions contain both LiCl and NaCl, we refer to them simply as High, Intermediate, and Low LiCl throughout the paper as reference to the corresponding concentration/dose of the toxic (LiCl) component of the stimulus. All solutions were presented at room temperature.

### Apparatuses

***Single-bottle training and test sessions*** were conducted in six identical Polycarbonate cages for *Experiments 1a and 2*. A stainless steel plate with a small slot, located on the center of the front wall of each cage, permitted access to an externally mounted sipper spout connected to a 100 ml graduated cylinder. An electrical contact circuit passing no more than 50 nA current through the animal was used to measure licks; each lingual contact with the stainless-steel spout completed the circuit. Time-stamped records of these contacts were saved for subsequent analyses (see below). Each cage was associated with its own 10 ml syringe and external programmable infusion pump, which were used to covertly administer solutions directly into the duodenum through tubing that ran from the external syringe that was routed through a stainless steel swivel connected to the ID catheter via the Luer Lok port in the rat’s harness. Opaque dividers were placed in between each cage to preclude observational learning/responding.

For Experiment 1b, single-bottle training sessions were conducted in a Davis Rig (Med Associates, St. Albans, VT). This apparatus comprised a wire mesh grid floor, three Plexiglas walls, and a stainless steel front wall. An access slot to the sipper tube was located in the center of the front wall, approximately 2 mm above the grid floor. The access slot was opened and closed by a computer-controlled shutter on the exterior of the front wall. The Davis Rig was outfitted with an infusion pump and line, as described above.

***Taste reactivity (TR)*** habituation and testing were conducted in a cylindrical chamber with clear Plexiglas walls and a clear Plexiglas floor for *Experiment 1a*. A mirror was mounted at a 45° angle just below the chamber floor and a digital video camera (Sony DSC-WX50 HD) was positioned facing the mirror on a tripod ~ 35 cm away. The infusion line, comprised of Tygon and Silastic tubing, ran from a 10 ml syringe (BD) in an external infusion pump (Harvard Apparatus) through a single channel stainless steel swivel (21G, Instech Solomon) mounted in the TR chamber lid through a spring tether for connection to the rat’s IO cannula. This arrangement allowed the rat to move freely about the chamber and prevented the rat from accessing the infusion line tubing. The TR chamber used in Experiment 1b was similar, except that the wide angle camera (Sony FDR-X3000R HD) was mounted ~30 cm directly below the clear Plexiglas floor.

***Progressive ratio (PR)*** training and testing for *Experiment 2* took place in one of four identical gustometers. See Spector et al [25] for a description of the gustometer. Briefly the gustometer is a modified operant chamber, consisting of three equally spaced access slots on the front panel (left, center, right) and interfacing computer-controlled fluid stimulus delivery and lick response measurement systems. For the purposes *of Experiment 2*, only the left access slot was used; the other two slots were blocked with a stainless steel shutter. Positioned ~ 2 mm behind the left access slot was a stationary Teflon Polytetrafluorethylene (PTFE) ball, which contained a stainless steel hollow hub (17G) for delivering fluids. Each lick at the PTFE ball was registered by a load cell. Depending on a predetermined schedule of reinforcement (see below), these licks could be programmed to operate a stepping motor, which was connected to an externally-mounted syringe, to dispense fluid through a polyethylene tube connected to the stainless steel hub.

### Behavioral procedures

#### Experiments 1a and 1b

All rats were first acclimated to the TR chamber and IO infusions of dH_2_O on each of three consecutive days. During these sessions, the rat was placed in the chamber and one of its IO cannulae was connected to the infusion line. Three minutes later the IO pump was started (1 ml/min) and ran for a total of 30-s starting from when the rat initiated oromotor responding. After the IO infusion, the rat was returned to the home cage. A pre-conditioning TR test session was conducted on the fourth day. This session was run identically to the acclimation sessions, except that 0.2% saccharin was infused in place of dH_2_O. Each rat was video recorded during this test session. The video records were later viewed offline for quantification of saccharin-elicited oromotor responses (details in *Data Analyses*). The rats were on ad libitum chow and water conditions in the home cage before and after these tests; no chow or water were present in the TR chamber itself.

One week after the pre-training TR test, all rats were placed on a restricted water-access schedule, in which dH_2_O was presented for 10 min each morning and for 30 min approximately 4–5 h later each afternoon. The morning sessions were conducted in the drinking cages (described above) and the afternoon sessions were conducted in the home cage. Rats were run in squads of six at a time. Immediately prior to the start of each session, the ID catheters were flushed with 0.5 ml of dH_2_O. Each rat was then connected to the ID infusion line and placed in the drinking cage. Once all six rats were ready, shutters covering the front access slots on each cage were removed, permitting access to the sipper spout and the intake session began. After 10 min elapsed, shutters were placed back over each access slot. Intake was measured to the nearest milliliter. On the first three of these sessions, the ID infusion pump was started immediately at the end of the intake session and run for 5 min (1 ml/min), but no ID infusate was delivered. This mock infusion was done to acclimate the rats to the sound of the pump. The rat was returned to its home cage after at the end of the mock infusion period. Then, beginning on the fourth day, a 5-min delay was introduced between the end of the intake session and the start of the ID infusion. Additionally, the rats received an ID infusion dH_2_O (15 ml/kg). Rats received two more such “water” sessions. The seventh day was the first CTA trial session. These sessions were run identically to the preceding “water” sessions, except that 0.2% saccharin was available at the drinking spout instead of dH_2_O and an ID stimulus was administered instead of dH_2_O. Rats were assigned to one of four ID stimulus groups in Experiment 1a. These groups were matched on the basis of previous day’s bodyweight, session 6 dH_2_O intake, and pre-training TR scores. In this experiment, the ID stimuli were a moderately high dose of LiCl (1.5 mEq/kg or 63.59 mg/kg, referred to here as High LiCl), a low dose of LiCl (0.15 mEq/kg or 6.36 mg/kg referred to here as Low LiCl), 18% lactose, and the isotonic NaCl control. The assigned ID stimulus was given at a volume of 15 ml/kg infused at a rate of 1 ml/min. As with the preceding water sessions, the ID infusion began 5 minutes after the end of the 10 min intake session. The ID infusion lines were thoroughly flushed with dH_2_O between each squad. Deionized water was presented as usual in the afternoon session.

On the next two days, rats received dH_2_O in the morning and afternoon sessions. Then, the 10^th^ day, a second conditioning trial was conducted in the morning session. This schedule (two “water” days, then a conditioning trial) continued for a total of six conditioning trials. These conditioning trials were run identically to the first one with the exception that if a rat failed to drink more than one milliliter (avoidance criterion) during a CTA trial, then 0.5 ml of the CS, 0.2% saccharin, was placed in its mouth via a 1 ml syringe, prior to the start of the ID infusion. This was done in order to ensure the rat associated the taste of the CS solution with the ID infusion. These rats were then given two “water” days like the rest of the rats, followed by a single-bottle test session. This single-bottle test session was run the same as a CTA trial, except that no ID infusion was given. The ID pump was run, but no infusate was administered. After the single-bottle test session, the rat was returned to its home cage as usual and its water bottle was returned during the afternoon session. These rats were discontinued from the training schedule and were given ad libitum access to water and chow for 6 days and then were given the post-training TR test.

Six days after the post-training single-bottle test (see below), all rats were re-acclimated to the TR chamber and IO infusion of dH_2_O in a single session as above. After the post-training TR test (described above), the rat was returned to its home cage and the two-bottle choice test was started. In this test, the rat received two bottles on the home cage, one contained 0.2% saccharin and the other contained dH_2_O. Twenty-four hours later, intake of each solution was measured (to the nearest ml), the bottles were replenished, and placed back on the home cage. The positions of the bottles were switched at that time to mitigate influence of side biases. Intake was measured again 24 h later and this concluded the two-bottle test.

Importantly, rats that met the avoidance criterion at any point during training after the first trial were immediately started on the test schedule. The number of days and procedures between the last conditioning trial and each test session were kept the same for all rats. If a rat consumed less than 1.0 ml on a conditioning trial, but then consumed more than one ml in the single-bottle test session, the appropriate ID infusate was administered and the rat was kept on the training schedule until it reached the avoidance criterion or until it had received six CTA trials, whichever came first. All rats that failed to meet the avoidance criterion by the end of six conditioning trials were discontinued from training and were run through the series of tests described above (single-bottle test, post-training TR, and two-bottle choice).

Experiment 1b was run identically to Experiment 1a, except the ID stimulus was an intermediate dose of LiCl (0.3 mEq/kg or 12.7 mg/kg) and intake was measured to the nearest 0.1 gram.

#### Experiment 2

Experiment 2 was conducted in a naïve set of rats. Before the taste-ID conditioning phase, all rats were trained and tested on the progressive ratio task as follows: home cage water bottles were removed approximately 20 h prior to the first training session. All rats were initially trained in the gustometers, in a single 30 min session, to obtain dH_2_O at the left access port by licking the response ball. Each lick delivered 5 μl of dH_2_O via the fluid delivery tube (continuous reinforcement schedule). After this and all other operant sessions terminated, the rats were returned to their home cages, without access to water, unless noted otherwise. On the following day, rats were again placed in the gustometer, but this time, access to fluid was attainable on a fixed ratio (FR) 3 schedule, whereby the rats had to perform 3 dry licks at the left response ball to earn 15 licks of dH_2_O (5 μl/lick over a period of 5 s). The session ended when the rat was inactive for a period of 5 min or 1.0 h had elapsed, whichever came first. A progressive ratio (PR) requirement was instituted on the third and fourth training days. On this schedule, the rat initially had to perform 1 dry lick to earn the same reward (15 licks/5 s of dH_2_O). The contingency increased by a factor of 1 on each successive trial to receive the same reward (i.e., PR 1). The session ended after 8 min of inactivity or 1 hr elapsed, whichever came first. On days 5–7, a PR2 schedule was in place. On this schedule and all schedules thereafter, the first ratio was set to 1 dry lick in order to give the rats a warm-up trial. The ratio increased by 2 for each successive trial (e.g., 1 dry lick, 2 dry licks, 4 dry licks, 6 dry licks, etc.). The time allowed to take the reward after reaching the dry lick criterion on each trial was extended to 3 min (15 licks; 5 ul/lick; 3-min) and the inactivity limit to end the session was reverted back to 3-min. A PR3 schedule was implemented in the 8^th^ training session. After this, water bottles were returned on the home cage and rats were given 8 days to recover to their ad lib bodyweights. Water bottles were once again removed and the rats were given a single PR3 refresher session, which was identical to the session prior to the break, except that the time to attain the water reward (15 licks) was shortened to 5 s. On the very next day, the test session was conducted to determine the rat’s unconditioned willingness to work to obtain saccharin solution on a PR3 schedule. Breakpoint was defined as the number of dry licks performed on the final reinforced ratio. Water bottles were returned on the home cage for repletion after this test session.

Two days later, home cage water bottles were removed and rats were placed on a restricted water access schedule for taste-ID training, just as described for Experiment 1. This phase, including the final single-bottle test, was run as in Experiment 1, except that it was extended to include a total possible 8 taste-ID conditioning trials. Rats were given ad lib chow and water in the home cage for six days after the single-bottle test. Water bottles were removed from the home cage on the sixth day and rats were re-acclimated to the PR3 test the following day with dH_2_O serving as the fluid reinforcer. On the very next day, a post-training PR3 test was conducted with 0.2% saccharin serving as the fluid reinforcer, in place of dH_2_O. Water bottles were returned on the home cage approximately 30 min after the test. Twenty four hours later, the home cage two-bottle test began.

### Data analysis

#### Single bottle training and testing sessions

CS intake on the first and last conditioning trial were compared in a repeated measures ANOVA, where group and/or trial were factors. Post hoc one-way ANOVAs and paired sample t-tests were used to break down the interaction. Similarly, CS intakes on the single-bottle test and two-bottle test and CS preference scores [CS intake/ (CS intake + dH_2_O intake)] were analyzed with separate one-way ANOVA and independent samples t-tests (Experiments 1a and 2). A Bonferroni correction factor was applied to account for multiple comparisons.

#### Microstructural analysis of licking on the last training session

Due to equipment malfunction, complete lick records were only available for the last training trial of *Experiment 2*. These were used to calculate total licks, average lick burst size per session, average number of bursts per session, and initial lick rate (i.e., total licks per the first min of the session, calculated from the start time of the second lick). Based on previous research, a pause criterion of ≥ 1 s was set to determine the end of a licking burst and the start of another [26]. Burst size was determined by tallying the total number of licks within the burst.

#### Taste reactivity

Video files were viewed off-line and in slow motion (frame by frame) by a trained observer, who was unaware of the stimulus or group assignment of the rat, using Sony Movie Studios HD software (v. 11). For each video, oromotor responses were categorized and quantified for the 30-s period that began with the first response. These responses have been described in detail elsewhere [23]. Ingestive responses included tongue protrusions, mouth movements, paw licks, and lateral tongue protrusions. Paw licking counts were derived by multiplying the time spent paw licking in each 30-s video by 6. Aversive responses included gapes, chin rubs, forelimb flails, and headshakes. Total ingestive and aversive responses were analyzed separately with one-way ANOVAs, using group as the between- subjects factor in Experiment 1a. Where appropriate, independent samples T-tests were used to make pairwise comparisons among groups. For Experiment 1a and 1b, separate paired samples T-tests were also conducted for each group to assess difference in total ingestive or total aversive responses from pre- to post-conditioning. A Bonferroni correction factor was used to account for multiple comparisons. P ≤ 0.05 was considered statistically significant for these and all subsequent analyses.

#### Progressive ratio

Pre- and post-conditioning breakpoints were compared within each training group with a Wilcoxon matched pairs test and across training groups using a Kruskal-Wallis test. These were followed by post hoc Mann-Whitney U tests, where appropriate. A Bonferroni correction factor was used to account for multiple comparisons. Additionally, a) the rate of operant responding, as indexed by the mean interval between each dry lick, b) rate of reinforcer intake, as indexed by the mean interval between the reinforcement licks taken, and c) the amount of reinforcer consumed (in total licks) were all separately plotted across each completed ratio for each individual rat.

## Results and discussion

### Experiment 1a

This experiment was designed to test the hypothesis that different visceral USs (LiCl versus Lactose) are capable of conditioning avoidance of the CS, as measured in intake tests, but only some visceral events (high LiCl) produces a concomitant change in CS-elicited oromotor reactions. Such results would indicate that taste avoidance and aversion are at least partially dissociable processes.

#### Training sessions

**Fig 1:**
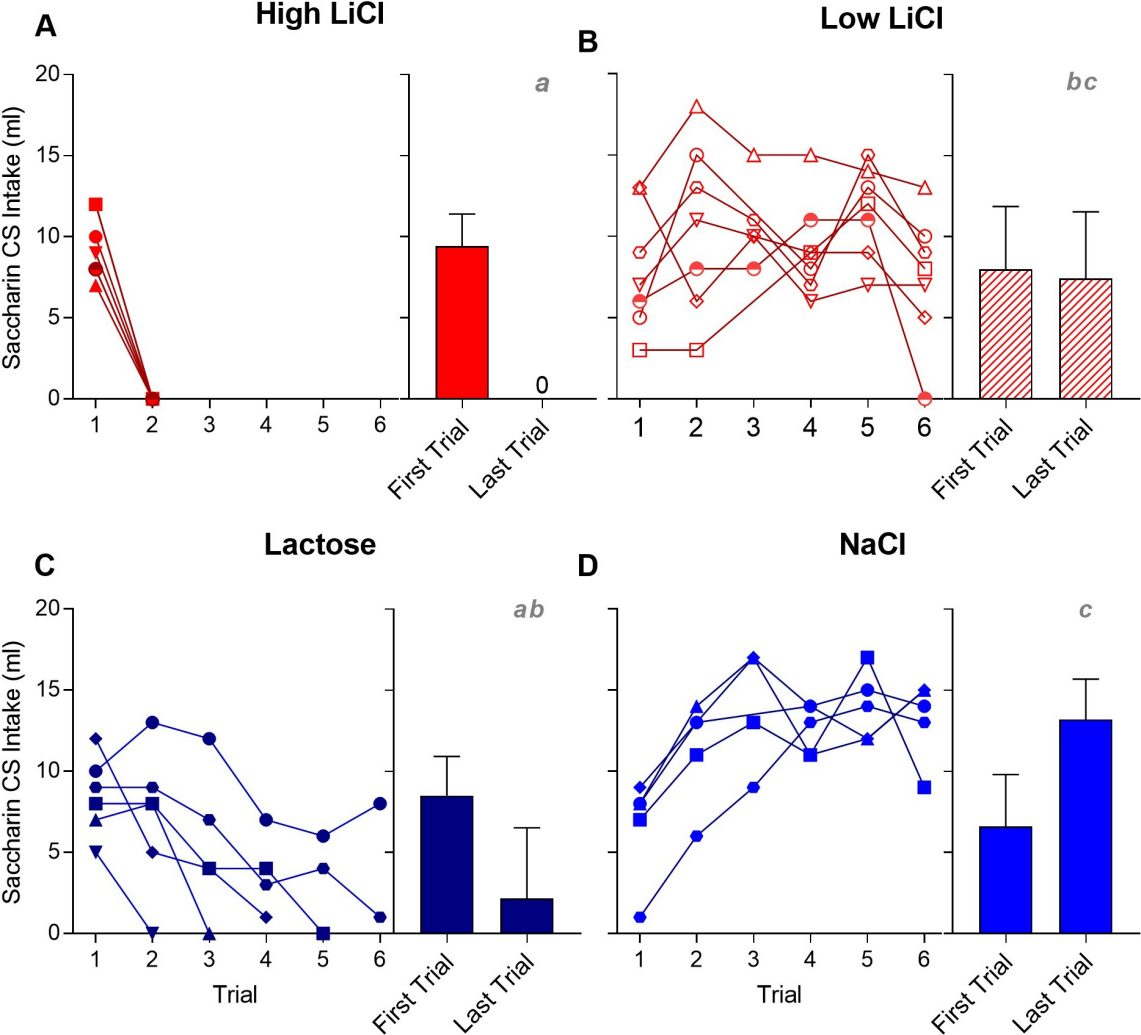
Saccharin CS intake is plotted across taste-ID conditioning trials for all individual rats within each training group [High LiCl, **A**; Low LiCl, **B**; Lactose, **C**; NaCl, **D**] in Experiment 1a. Group mean ± SEM saccharin CS intake on the first and last training trials is shown to the right of the individual plots. A given rat in each training group is represented by the same symbol across all Experiment 1a figures. Histograms with different gray letters were found to be significantly different from one another with post hoc t-tests, Bonferroni-corrected for multiple comparisons.

**Fig 2:**
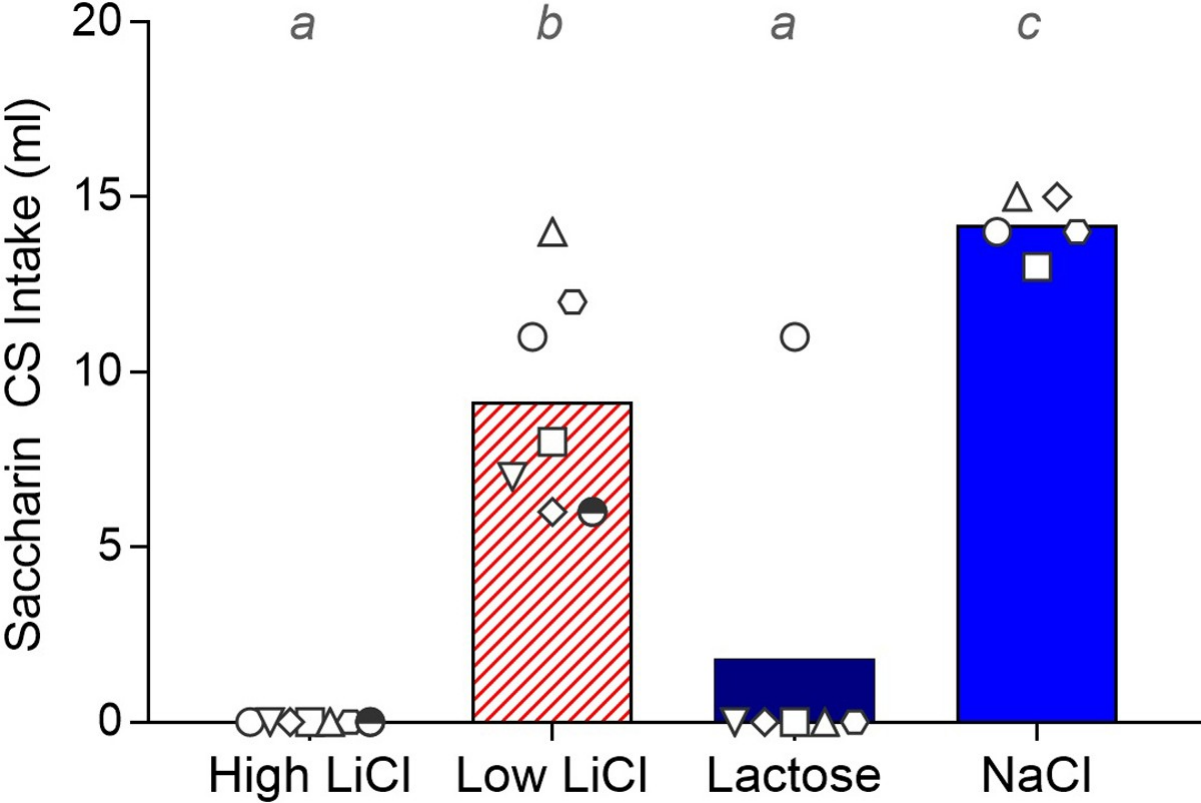
Filled histograms show mean total intake of the saccharin CS on the single-bottle test for each training group in Experiment 1a after taste-ID conditioning, with the total intake of each individual rat within each training group indicated by the white symbols. A given rat in each training group is represented by the same symbol across all Experiment 1a figures. Histograms with different gray letters were found to be significantly different from one another with post hoc t-tests, Bonferroni-corrected for multiple comparisons.

Overall, all four training groups drank comparable amounts of the saccharin CS on the very first trial of training (see Fig 1, with statistics in Table 1). Fig 1 shows that after just one trial, all of the High LiCl rats and one of the Lactose rats completely avoided the saccharin. Most of the Lactose rats eventually ceased consuming the CS (within an average of 4 trials), with the exception of one rat that continued to drink saccharin despite receiving an equivalent dose per bodyweight of lactose. None of the Low LiCl rats consistently avoided the saccharin CS across training; instead, they consumed roughly the same amount across all training trials. One Low LiCl rat seemingly avoided the saccharin on the sixth (final) trial, but, as can be seen in Fig 2, this same rat consumed on par with its usual intake in the subsequent single-bottle test. The NaCl control group, on the other hand, significantly increased intake of the saccharin CS across training.

**Table 1:** Statistical outcomes for Experiment 1.

**First versus Last Taste-ID Conditioning Trial Intake**			
	Group	Trial	Group x Trial
1^st^ versus Last Trial	*F(3*, *21) = 6*.*31*, *p = 0*.*003*	*F(1*, *21) = 12*.*51*, *p = 0*.*002*	*F(3*, *21) = 22*.*84*, *p = 0*.*000001*
1^st^ Trial Only	F(3, 21) = 0.92, p = 0.45	N/A	N/A
Last Trial Only	*F(3*, *21) = 25*.*63*, *p <0*.*000001* ^*a*^	N/A	N/A
**Single-Bottle Intake Test**			
Group Effect	*F(3*, *21) = 32*.*38*, *p <0*.*000001* ^*a*^		
**Ingestive Taste Reactivity Scores**			
	Group	Test (Pre v. Post)	Group x Test
Pre- v. Post-Conditioning	*F(3*, *21) = 6*.*18*, *p = 0*.*004*	*F(1*, *21) = 6*.*32*, *p = 0*.*02*	*F(3*, *21) = 6*.*56*, *p = 0*.*003*
Pre-Conditioning Only	F(3, 21) = 0.24, p = 0.87	N/A	N/A
Post-Conditioning Only	*F(3*, *21) = 9*.*09*, *p = 0*.*0005* ^*a*^	N/A	N/A
**Aversive Taste Reactivity Scores**			
	Group	Test (Pre v. Post)	Group x Test
Pre- v. Post-Conditioning	*F(3*, *21) = 35*.*51*, *p < 0*.*000001*	*F(1*, *21) = 44*.*47*, *p <0*.*000001*	*F(3*, *21) = 35*.*82*, *p < 0*.*000001*
Pre-Conditioning Only	F(3, 21) = 1.13, p = 0.36	N/A	N/A
Post-Conditioning Only	*F(3*, *21) = 35*.*65*, *p < 0*.*000001* ^*a*^	N/A	N/A
**Two-Bottle Choice Test (Preference Score)**			
Group Effect	*F(3*, *21) = 16*.*58*, *p = 0*.*00001* ^*a*^		

Notes. Statistically-significant outcomes are italicized.

^a^ Post hoc comparisons are indicated in the corresponding figure, where appropriate.

#### Post-training single-bottle test

As expected, the NaCl group consumed more of the CS than the High LiCl, Low LiCl, and Lactose groups in the single-bottle test session (see Fig 2 and statistics in Table 1). In fact, all of the rats in the High LiCl and, with the exception of one rat, all of the rats in the Lactose group completely avoided the CS. On average, the Low LiCl group consumed significantly more than the High LiCl and Lactose groups, though intake was notably quite variable in this group. As shown in Fig 2, approximately half of the Low LiCl rats consumed a moderate amount of the CS, while the remaining half consumed nearly as much as the rats in the NaCl group did.

#### Pre- and post-training taste reactivity tests

**Fig 3:**
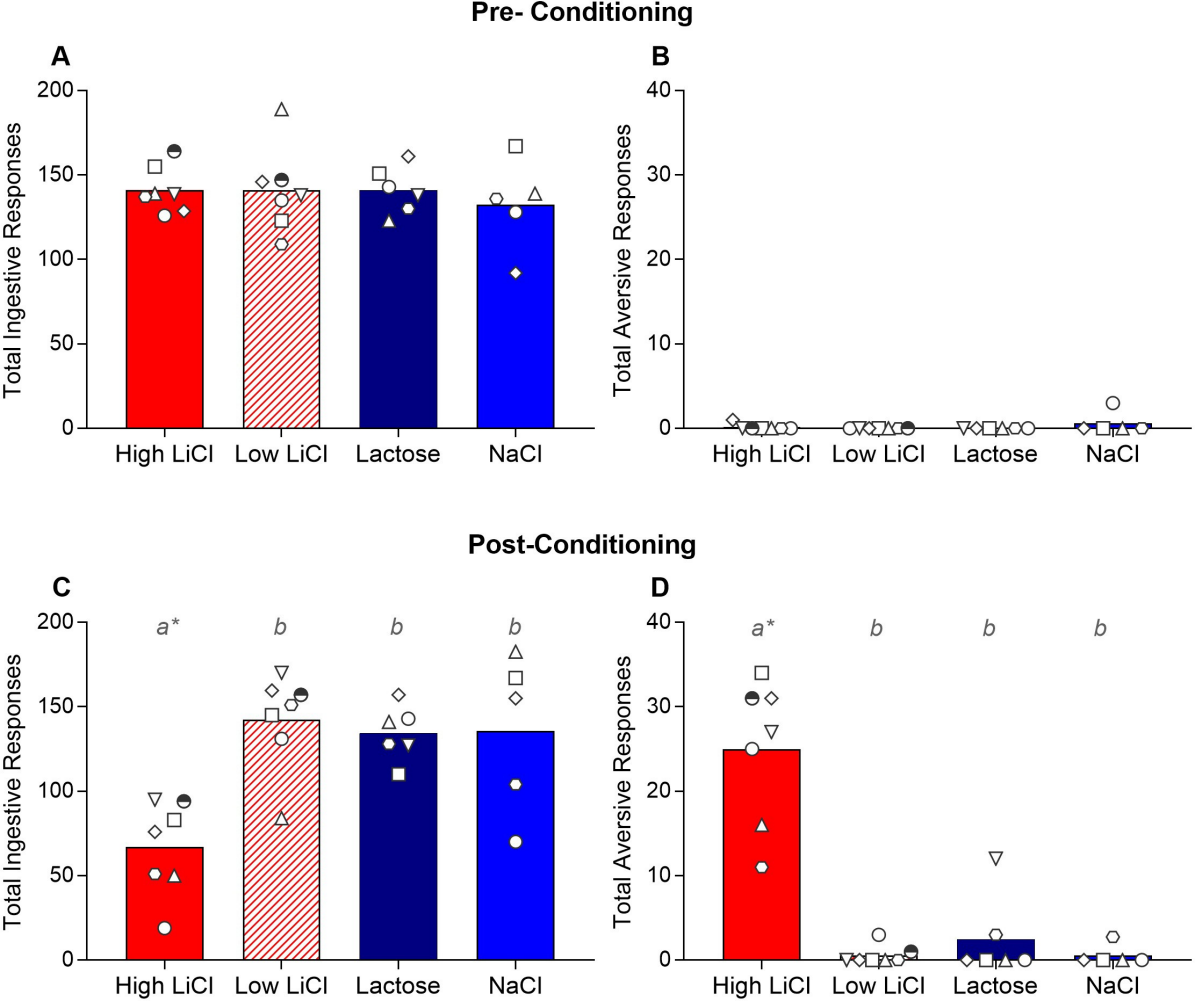
Top panel: Filled histograms show mean total ingestive (A) and aversive (B) taste reactivity responses elicited by a brief intraoral infusion of saccharin, the CS, prior to taste-ID conditioning, for each of the four training groups from Experiment 1a. Bottom panel: Filled histograms show mean total ingestive (C) and aversive (D) taste reactivity responses elicited by a brief intraoral infusion of saccharin, the CS, after taste-ID conditioning for each training group from Experiment 1a. The responses of each individual rat within each training group are plotted in the white symbols. The symbol for a given rat within each group corresponds across graphs in this figure and other Experiment 1a figures. Histograms with different gray letters were found to be significantly different from one another with post hoc t-tests, Bonferroni-corrected for multiple comparisons. The asterisk indicates post-training score was significantly different from pre-training score, after Bonferroni correction.

Prior to training, all rats elicited primarily ingestive responses and virtually no aversive responses, with no statistically significant differences among training groups (see Fig 3A and 3B and Table 1). After training, however, the High LiCl rats showed a significant change in the TR profile. Specifically, the High LiCl rats exhibited a reduction in ingestive TR and a corresponding significant increase in aversive TR to intraorally infused saccharin (Fig 3C and 3D). Despite the fact that the Lactose group showed comparable reductions in CS intake by the end of training and in the single-bottle test, these rats did not exhibit a change in TR profile in the post-training TR test. In fact, on average, the Lactose group’s ingestive and aversive post-training TR scores were similar to that of the Low LiCl and the NaCl group. Consistent with this, only the High LiCl group showed significant changes in ingestive and aversive responses from pre- to post-conditioning (ps ≤ 0.0002; all other ps > 0.12).

#### Post-training two-bottle choice test

**Fig 4:**
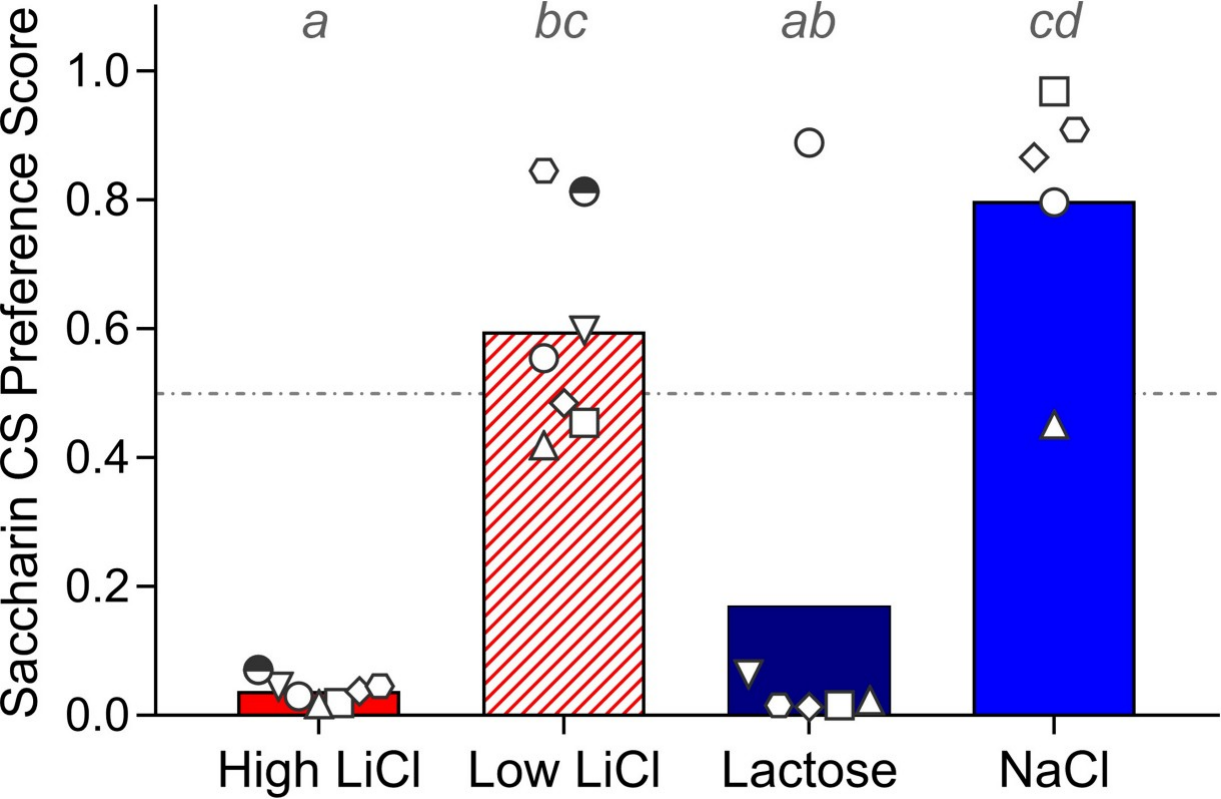
Filled histograms show mean post-conditioning preference for the saccharin CS on the two-bottle choice test for each training group for Experiment 1a, with the preference score of each individual rat within each training group indicated by the white symbols. A given rat in each training group is represented by the same symbol across all Experiment 1a figures. Histograms with different gray letters were found to be significantly different from one another with post hoc t-tests, Bonferroni-corrected for multiple comparisons.

Lastly, the CS was presented in a 48-hr choice test against dH_2_O on the home cage. As shown in Fig 4, the High LiCl and Lactose groups preferred dH_2_O to the saccharin CS. These preference scores were significantly lower than that of the NaCl control group, which generally preferred saccharin to dH_2_O. The Low LiCl group also showed preference for the CS, but it did not differ statistically from that of either the Lactose or NaCl groups.

### Experiment 1b

In order to interrogate whether the quality of the visceral stimulus is an critical determinant in the response profile conditioned, we included groups that received either a low or high dose of LiCl in Experiment 1a, with the expectation that low LiCl would foster a change in TR, even if it suppressed intake less expediently than a stimulus that produces lower GI distress (i.e., lactose). However, the low LiCl dose we selected was not sufficient to suppress intake, even after 6 trials. Thus, here, we replicated the basic paradigm in a group of naïve rats that received 0.2% saccharin paired an intermediate dose of LiCl (0.3 mEq/kg) ID infused.

**Fig 5:**
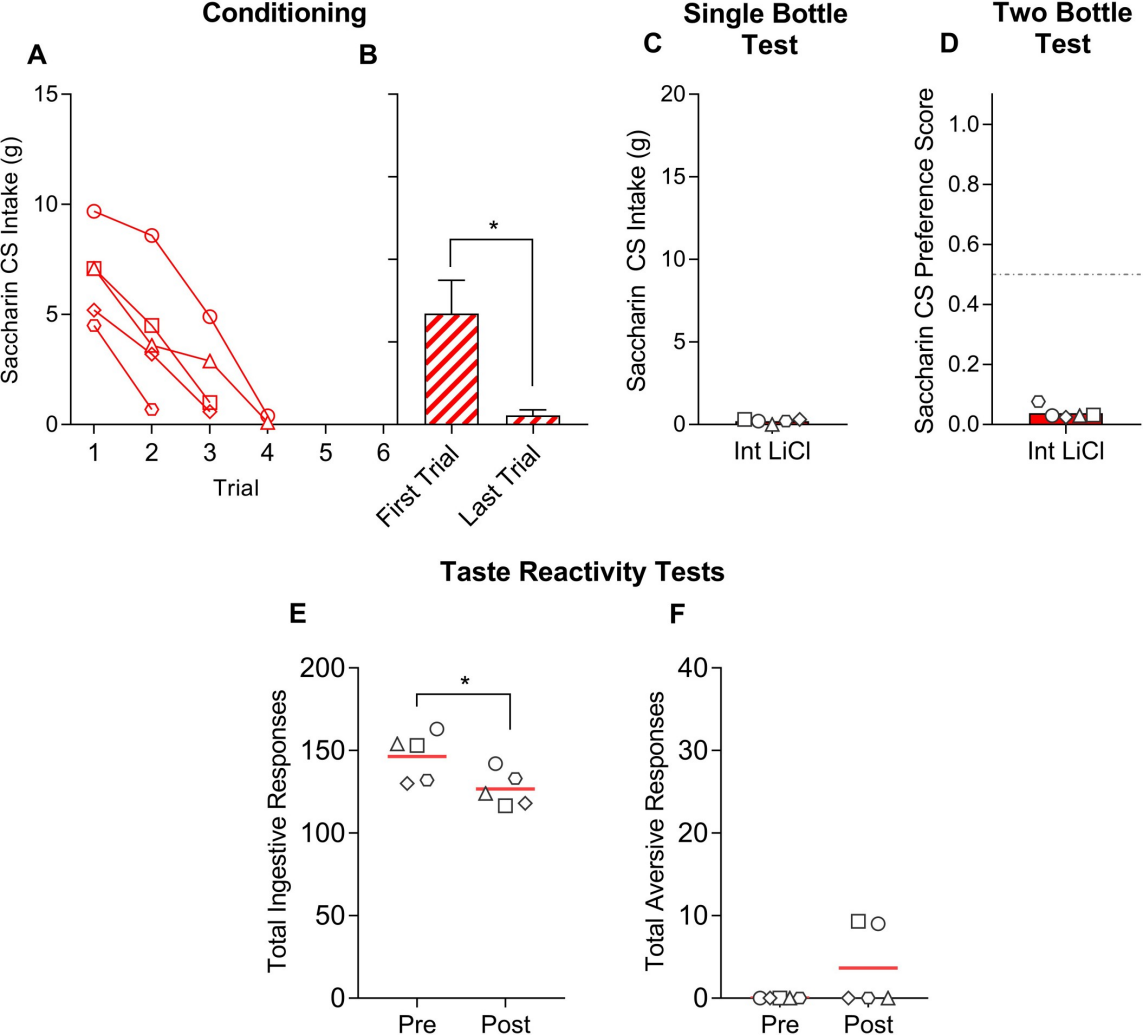
**A**: Saccharin CS intake is plotted across taste-ID conditioning trials for all individual rats in the Intermediate LiCl group of Experiment 1b. **B**: Group mean ± SEM saccharin CS intake on the first and last training trials [t(4) = 6.39, p = 0.003]. **C** and **D**: Filled histograms show mean total intake of the saccharin CS on the single-bottle test and mean saccharin preference score, respectively, after saccharin-ID Intermediate LiCl conditioning. **E** and **F**: Filled histograms show mean total ingestive and aversive taste reactivity responses elicited by a brief intraoral infusion of saccharin, the CS, before and after taste-ID Intermediate LiCl conditioning, respectively [Ingestive pre versus post: t(4) = 2.98, p = 0.04; Aversive pre versus post: t(4) = 1.63, p = 0.18]. In each panel, the white symbols represent individual rats and the same symbol is used for a given rat across all panels. Asterisks indicate significant differences.

The Intermediate LiCl group gradually reduced CS intake across conditioning, such that intake on the final trial was significantly lower than that on trial 1 (see Fig 5A, with statistics in corresponding figure caption). On average, these rats avoided the CS within ~ 3 trials and subsequently avoided the CS in the single-bottle test and two-bottle choice test (see Figs 4B and 5C and 5D, respectively). Taste reactivity to the saccharin CS was measured before and after conditioning, as in Experiment 1a. Despite clear avoidance of the CS in intake tests, rats exposed to the intermediate dose of LiCl did not significantly increase aversive responses from the pre- to post-conditioning TR test. They did, however, show a modest, but significant, reduction in ingestive TR from the pre- to post-conditioning test (see Fig 5E–5F).

### Experiment 2

Provided ID lactose conditioned avoidance of the saccharin CS in Experiment 1, without affecting saccharin-elicited TR, we hypothesized that this learning would impact other motivational effectors, namely appetitive responses, geared at obtaining the saccharin CS. Thus, before and after taste-ID training, rats were tested for their willingness to work to obtain access to saccharin solution, in a PR test. Moreover, we assessed whether the avoidance produced by High LiCl and Lactose was also associated with distinguishing patterns of licking behavior towards the saccharin CS, during the final taste-ID training session.

#### Training sessions

**Fig 6:**
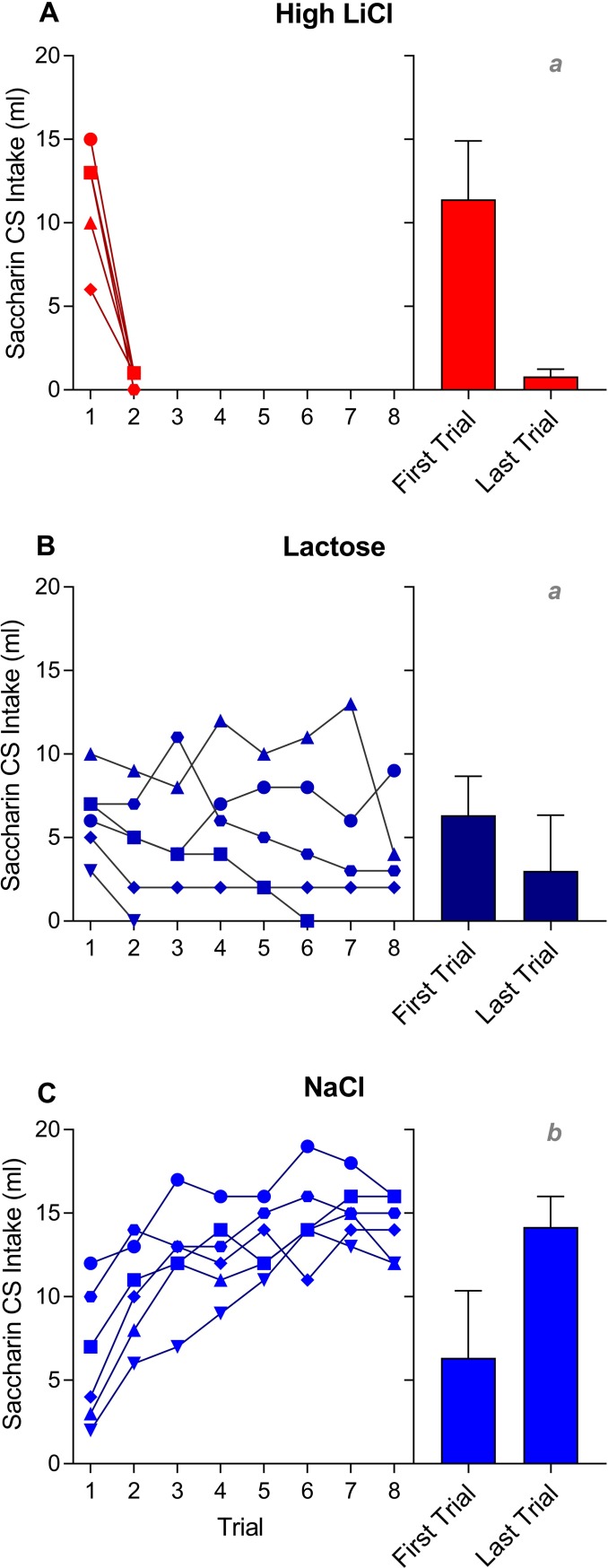
Saccharin CS intake is plotted across taste-ID conditioning trials for all individual rats within each training group [High LiCl, **A**; Lactose, **B**; NaCl, **C**] in Experiment 2. Group mean ± SEM saccharin CS intake on the first and last training trials is shown to the right of the individual plots. A given rat in each training group is represented by the same symbol across all Experiment 2 figures. Histograms with different gray letters were found to be significantly different from one another with post hoc t-tests, Bonferroni-corrected for multiple comparisons.

Just as in Experiment 1, all groups consumed considerable amounts of saccharin on the very first trial of training (see Fig 6). The High LiCl group completely avoided the saccharin CS by trial 2. As before, the NaCl control group increased intake across training, such that by the end of training total CS intake was significantly higher for the NaCl group, as compared to the High LiCl group and the Lactose group. That said, only two of the Lactose rats learned to completely avoid the CS. Despite extensive training (this time, 8 trials), out of the remaining four Lactose rats, three maintained a low level of CS intake and one consumed relatively high amounts of the CS.

#### Microstructural analyses of licking on the last trial

**Fig 7:**
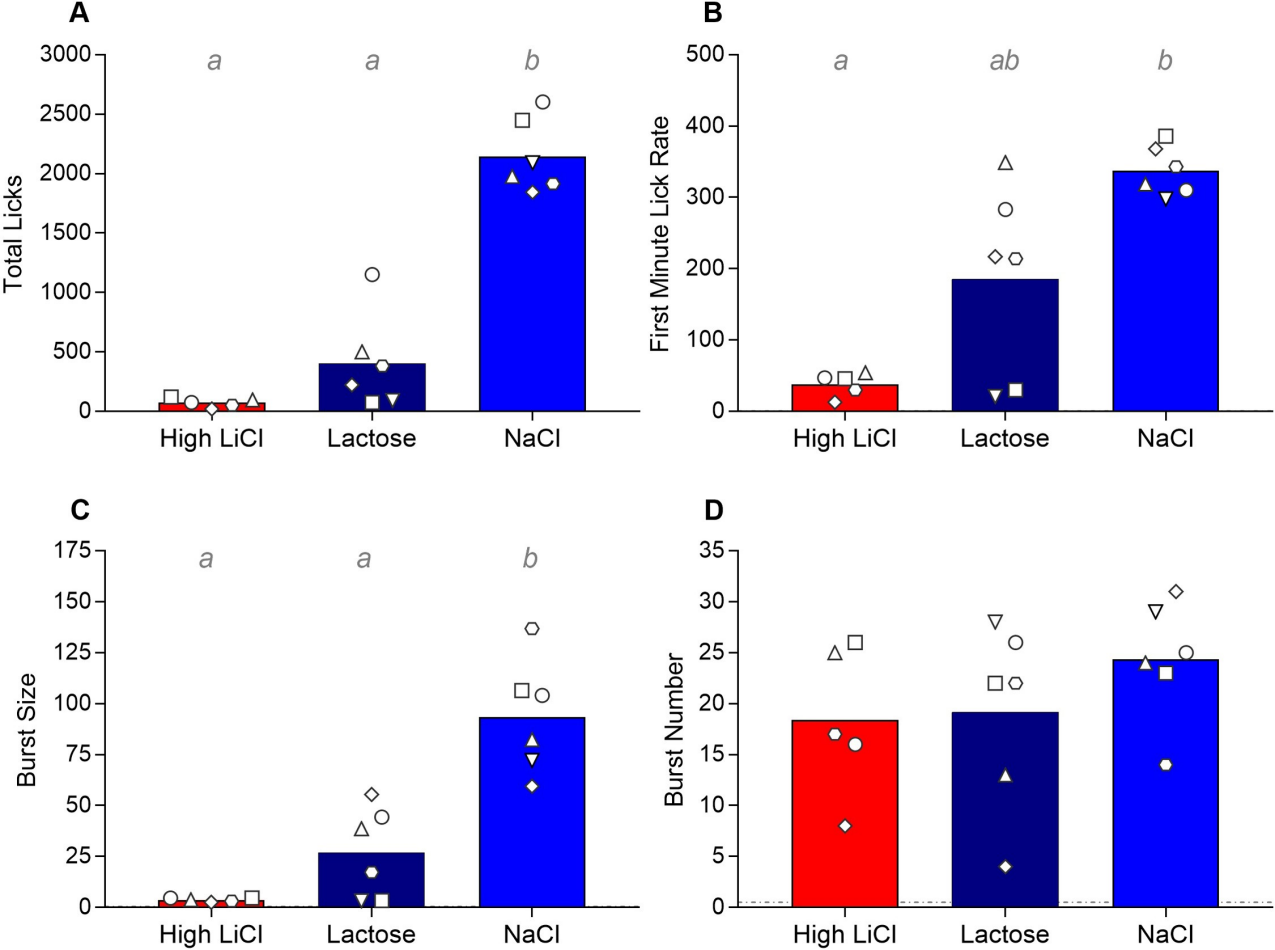
Filled histograms show mean total licks (**A**), lick rate in the first minute of the session (**B**), overall lick burst size (**C**) and lick burst number (**D**) for the saccharin CS on the final taste-ID conditioning trial for each training group for Experiment 2, with each individual rat within each training group indicated by the white symbols. Note that each rat within a training group is represented by the same symbol shape in all graphs for Experiment 2. Histograms with different gray letters were found to be significantly different from one another with post hoc t-tests, Bonferroni-corrected for multiple comparisons.

During the first minute of the final training trial, the High LiCl group took very few licks, significantly less than the NaCl control group (see Fig 7B and Table 2). The Lactose group took an intermediary number of licks in the first minute, such that this group did not significantly differ from either the High LiCl group or the NaCl group. There were very divergent responses in the Lactose group. That is, the two rats that discontinued drinking the saccharin CS looked similar to the rats in the High LiCl group, whereas the remaining Lactose rats took a few hundred licks in the first minute. Looking at the entire 10-minute session, all groups took statistically similar number of bursts (Fig 7D). Thus, the relatively little amount of the saccharin CS that the High LiCl and Lactose groups drank on the last trial was taken in many small bursts. Both groups had significantly smaller bursts than did the NaCl group.

**Table 2:** Statistical outcomes for Experiment 2.

**First versus Last Taste-ID Conditioning Trial Intake**				
	**Group**	**Trial**		**Group x Trial**
1^st^ versus Last Trial	*F(2*, *14) = 8*.*93*, *p = 0*.*003*	*F(1*, *14) = 6*.*53*, *p = 0*.*02*		*F(2*, *14) = 44*.*84*, *p < 0*.*00001*
1^st^ Trial Only	*F(2*, *14) = 4*.*02*, *p = 0*.*04* ^*b*^	N/A		N/A
Last Trial Only	*F(2*, *14) = 55*.*89*, *p < 0*.*00001* ^*a*^	N/A		N/A
**Microstructural Aspects of Licking on the Final Taste-ID Conditioning Trial**				
	**Group Effects**			
Total Licks	*F(2*, *14) = 77*.*08*, *p < 0*.*000001* ^*a*^			
1^st^ Minute Lick Rate	*F(2*, *14) = 17*.*82*, *p = 0*.*0001* ^*a*^			
Burst Size	*F(2*, *14) = 27*.*02*, *p = 0*.*00002* ^*a*^			
Burst Number	*F(2*, *14) = 1*.*05*, *p = 0*.*38*			
**Single Bottle Intake Test**				
Group Effect	*F(2*, *14) = 87*.*27*, *p < 0*.*00001* ^*a*^			
**Pre-Conditioning Progressive Ratio Breakpoint**			**Post-Conditioning Progressive Ratio Breakpoint**	
H(2, n = 17) = 0.57, p = 0.75			*H(2*, *n = 17) = 7*.*32*, *p = 0*.*03* ^*a*^	
**Two-Bottle Choice Test (Preference Score)**				
Group Effect	*F(2*, *14) = 26*.*88*, *p = 0*.*00002* ^*a*^			

Notes. Statistically-significant outcomes are italicized.

^a ^Post hoc comparisons are indicated in the corresponding figure, where appropriate.

^b^ None of the post hoc comparisons were significant after Bonferroni correction.

#### Post-training single-bottle test

**Fig 8:**
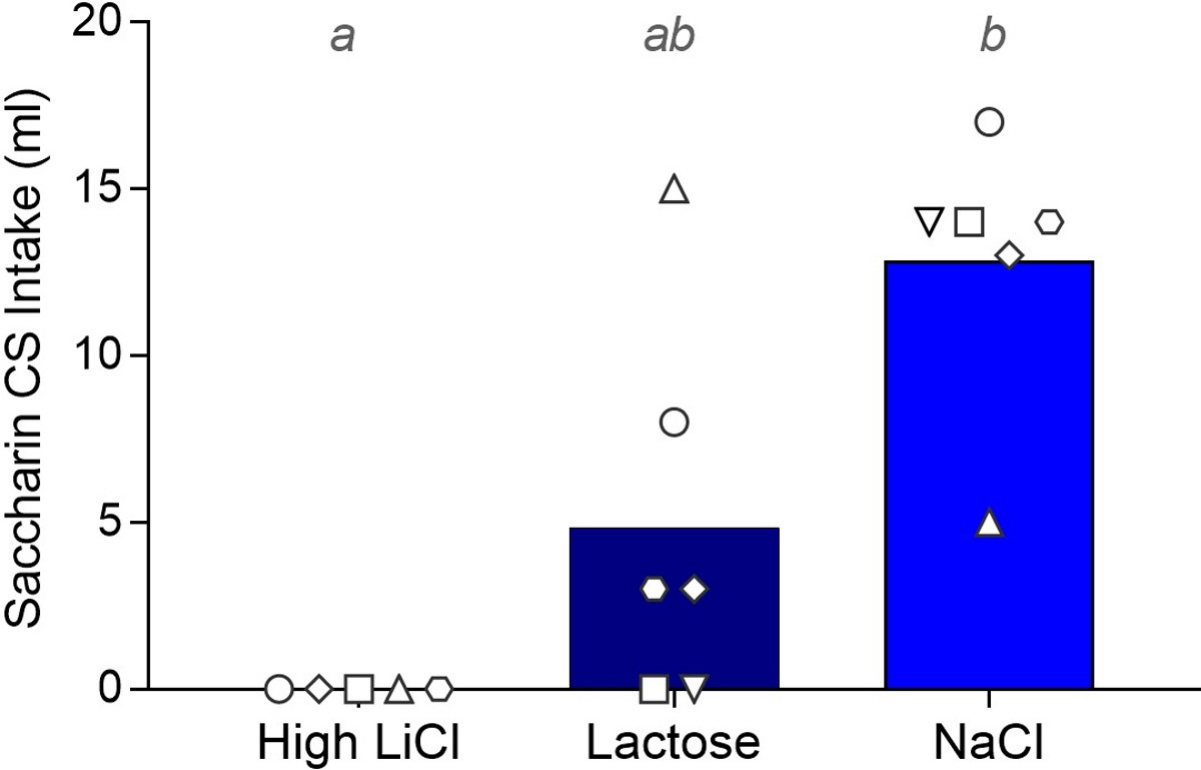
Filled histograms show mean total intake of the saccharin CS on the single-bottle test for each training group after taste-ID conditioning for Experiment 2, with the total intake of each individual rat within each training group indicated by the white symbols. A given rat in each training group is represented by the same symbol across all Experiment 2 figures. Histograms with different gray letters were found to be significantly different from one another with post hoc t-tests, Bonferroni-corrected for multiple comparisons.

Intake on the post-training single-bottle test looked very similar to the last trial of training (see Fig 8). The High LiCl group consumed significantly less than the NaCl group. On average, the Lactose rats consumed an intermediary amount, not statistically different from either the High LiCl or the NaCl group. Intake in the Lactose group was highly variable, with two rats essentially avoiding the CS, two rats consuming limited amounts of the CS, and two rats consuming fairly substantial amounts of the CS.

#### Pre- and post-training progressive ratio tests

**Fig 9:**
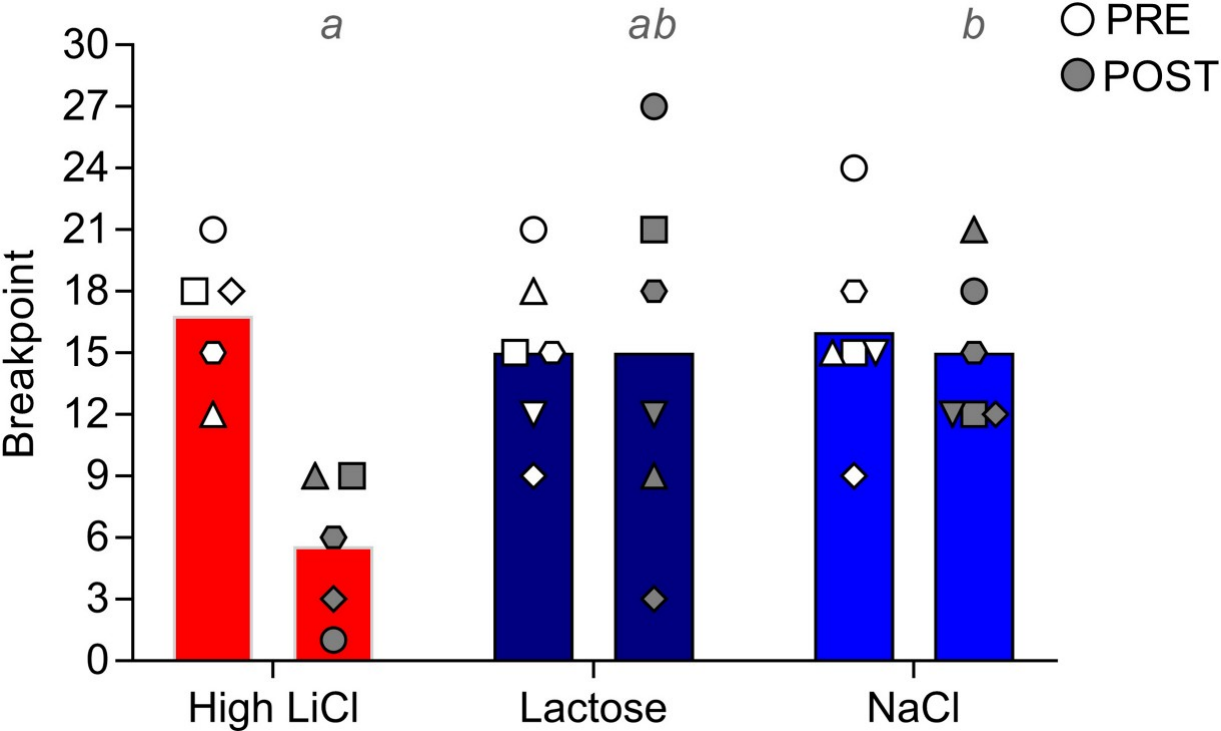
Filled histograms show median breakpoint for the saccharin reinforcer before and after taste-ID conditioning for the three training groups in Experiment 2. The breakpoints by the individual rats within each training group are indicated in the symbols. A given rat in each training group is represented by the same symbol across all Experiment 2 figures. Post-conditioning histograms with different gray letters were found to be significantly different from one another with post hoc Mann-Whitney U tests, Bonferroni-corrected for multiple comparisons.

Fig 9 shows the pre- and post-conditioning median breakpoints for the saccharin CS; the corresponding statistical outcomes are in Table 2. All groups had comparable breakpoints prior to taste-ID training. After training, the High LiCl had a significantly diminished breakpoint, compared to the NaCl group. The Lactose group did not exhibit a post-conditioning decrease in breakpoint for saccharin, relative to the NaCl group. Post-conditioning breakpoint also did not differ between the High LiCl and Lactose groups; however, this comparison just missed the cut-off for statistical significance (p = 0.056, uncorrected). Interestingly, the two Lactose rats that completely avoided the saccharin CS by the end of training and in the single-bottle test either increased or did not change their operant responding for the same stimulus. Despite the fact that many of the NaCl rats actually increased intake across taste-ID training, this did not translate to an increase in the willingness to work for saccharin during the post-training progressive ratio test.

#### Post-training two-bottle choice test

**Fig 10:**
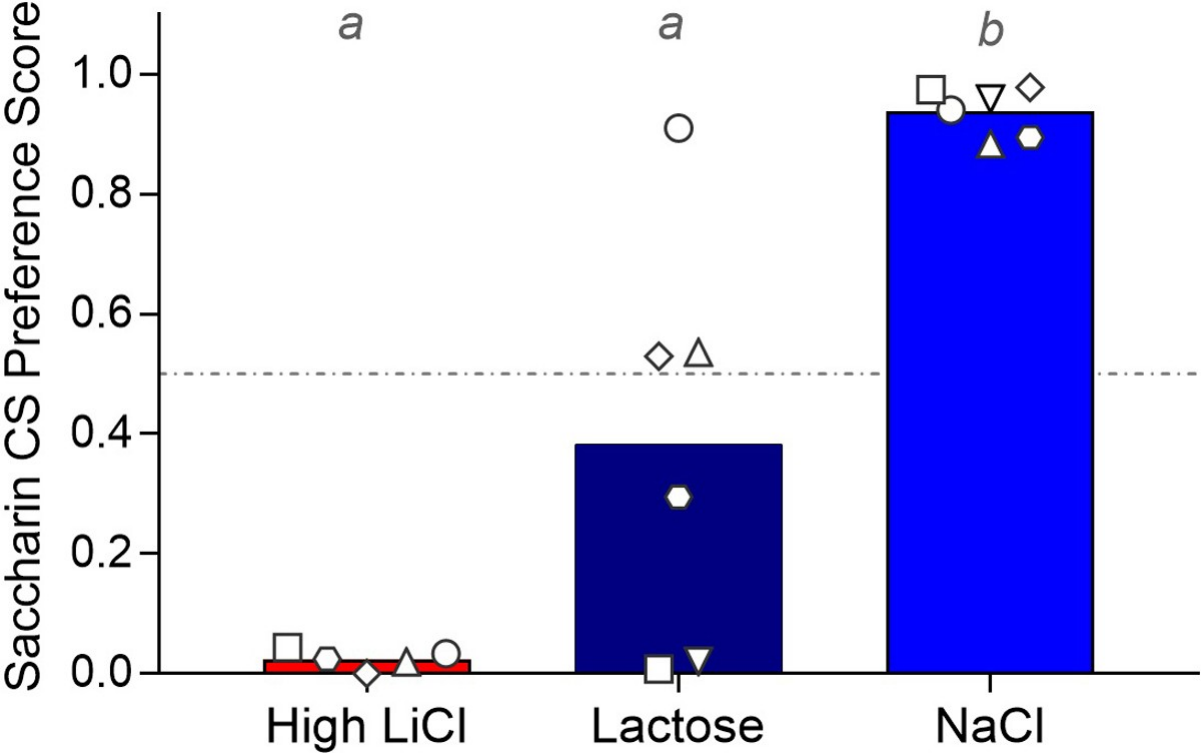
Filled histograms show mean post-conditioning preference for the saccharin CS on the two-bottle choice test for each training group for Experiment 2, with the preference score of each individual rat within each training group indicated by the white symbols. A given rat in each training group is represented by the same symbol across all Experiment 2 figures. Histograms with different gray letters were found to be significantly different from one another with post hoc t-tests, Bonferroni-corrected for multiple comparisons.

In the subsequent two-bottle test, the High LiCl rats completely avoided the saccharin, preferring dH_2_O instead, and the NaCl group exhibited near total preference for saccharin (see Fig 10 and Table 2). The Lactose group exhibited significantly lower CS preference, compared to the NaCl control group. The rats that avoided the CS in the training and single-bottle test also avoided it in the two-bottle test.

S2 Fig displays the responses of each individual rat in each of the three training groups across the intake, licking microstructure, preference, and PR tests. Although there is variability in the conditioned responses to ID lactose in this relatively small cohort, this summary figure indicates that for a given rat, a conditioned suppression in intake of a taste CS paired with ID lactose (top four panels) does not necessarily negatively impact appetitive responding for small volumes of that same solution in the PR test (lower panel).

## General discussion

Consistent with previous work [14, 22], we found that while High LiCl (1.5 mEq/kg), intermediate LiCl (0.3 mEq/kg) and lactose all conditioned a decrease in CS intake and preference—what some term conditioned taste avoidance—only High LiCl conditioned a concomitant increase in aversive oromotor reactivity—i.e., conditioned taste aversion. Moreover, here we show that this dissociation extends to another domain of taste function. Namely, CS-High LiCl associations reduced appetitive responding for the taste CS, while CS-ID Lactose associations had no such effect on the subsequent willingness to work for the same CS, in a PR task. Thus, together, the results of these experiments provide compelling evidence that learned taste avoidance is not necessarily accompanied by a change in oromotor consummatory reactions evoked by the associated taste solution (i.e., CS) or appetitive behaviors geared towards obtaining the CS. One powerful feature of the design used here is that the postingestive consequences of the US was either absent or minimized during all of the test sessions. This allowed us to compare responses guided by the CS solution, without influence of the postoral US.

### High and intermediate LiCl

ID infusions of 1.5 mEq/kg (High) LiCl resulted in virtually no intake of the associated saccharin CS after just one training trial, as measured in single-bottle and two-bottle choice tests. Moreover, this experience with High LiCl significantly shifted the TR profile evoked by the associated taste CS such that ingestive responses were replaced with aversive, rejective responses after training. A lower dose of LiCl (0.3 mEq/kg; intermediate) likewise completely suppressed CS intake, but this required more conditioning trials than it did with the High LiCl dose. Intermediate LiCl did not result in a shift from ingestive to aversive responses to saccharin in the final TR test. Unlike the High LiCl group, all rats in the Intermediate LiCl group elicited principally ingestive responses to the saccharin CS after training. That said, ingestive responses in the Intermediate LiCl group did decrease by ~14% from pre- to post-conditioning; this could be indicative of a modest change in the consummatory domain. Experiment 2 further demonstrated that after two saccharin CS-High LiCl pairings, a separate group of rats significantly reduced their PR breakpoint for the taste CS, suggesting that the appetitive aspects of taste-guided behavior that underlie progressive ratio performance are also modified by experience with the associated visceroceptive consequences of High LiCl.

### Lactose

ID Lactose also produced a reduction in CS intake across training, though this outcome was considerably more variable than that produced by High LiCl. The gradual and varied avoidance of the CS across training displayed by the Lactose rats was remarkably similar to that of the Intermediate LiCl group. Some rats seemed to rapidly shut down CS intake (within just one or two trials), whereas other rats gradually reduced CS intake over a few trials. Other rats seemed unfazed by the ID lactose infusions and maintained high levels of intake across training and in subsequent tests. Generally speaking though, if a rat drank zero or near zero milliliters of saccharin at the end of training and in the single-bottle test, then it also avoided saccharin in the two-bottle test. However, even rats that completely avoided saccharin in these tests, did not evince a post-conditioning shift in TR to the CS like the High LiCl rats did. Rather these Lactose rats primarily showed ingestive responses to saccharin and little to no aversive responses. In fact, we note that two out of the three Lactose rats that expediently avoided the CS in conditioning, actually increased ingestive TR from the pre- to post-conditioning test. By contrast, the Intermediate LiCl rats, with the exception of one, all decreased their ingestive responses from pre- to post-conditioning. Overall, the changes in intake conditioned by ID lactose did not seem to be related to a fundamental change in the consummatory domain. Nor did this experience result in a reduction in operant responding for the taste stimulus in the PR test (Experiment 2), relative to the NaCl control group. Even the two rats that completely avoided saccharin intake in the single-bottle and two-bottle tests on Experiment 2 exhibited a willingness to work for saccharin at levels comparable to NaCl controls in the PR test.

### Low LiCl & NaCl

The 0.15 mEq/kg (Low) LiCl infusions after saccharin CS consumption (Experiment 1 only) did not change intake across training, but intake and preference were somewhat blunted relative to that of the NaCl group (albeit not statistically so). That is, the NaCl group tended to increase in intake as training progressed, whereas the Low LiCl group did not. Nevertheless, the Low LiCl, like the NaCl group and Lactose groups, exhibited mostly ingestive TR after training. It was somewhat surprising that the rise in CS intake in the NaCl group was not met by a concomitant increase ingestive TR (Experiment 1) or breakpoint (Experiment 2). One possibility is that the small volumes of saccharin achieved in the PR test is discounted after the same solution was achieved with less effort in the previous 10-minute free access sessions.

### Critical features of the visceral US as response determinants

With the bright noisy water experiment, Garcia and Koelling [13] demonstrated relatively early on that taste avoidance learning was subject to some degrees of associative and response selectivity. Namely, rats learned to avoid a flavor paired with a toxin or radiation more expediently than a flavor paired with a shock. Conversely, rats learned to avoid an audiovisual cue associated with shock over one paired with the toxin or radiation. The studies imply that the nervous system is organized in such a way to facilitate the association of certain sensory inputs with certain types of response outputs. The literature is now replete with examples of various types of interoceptive stimuli that effectively condition reductions in intake of the associated taste CS. This includes, in addition to the ones already mentioned, motion/vestibular disturbance, GI pain, bacterial infection, chemotherapeutic drugs, and certain drugs of abuse, among others [e.g., [8, 15, 27–33].

Based on their initial observations in humans [16], Pelchat et al [14] put forth the hypothesis that even within this subdomain of taste avoidance, the responses rendered depend on the particular *type* of visceral US. Although a number of studies have been published in the years since, showing that certain stimuli can condition avoidance without affecting aversive TR {e.g., [30, 32, 34–37] and for reviews, see [15, 31]}, the critical features of the visceral US that dictate the conditioned response, remain to be fully understood. Notably, peripheral blockade of 5-HT_3_ receptors with ondansetron and central depletion of serotonin both abolish LiCl-induced taste aversion, as measured by aversive TR, but does not interfere with the LiCl-induced taste avoidance, leading to the hypothesis that engagement of this serotonergic system is a determinant of aversion, but not avoidance [38, 39], though other features of the visceral stimulus may be sufficient. One thing that has been historically difficult to separate from US quality is US intensity. For example, in the aforementioned study disruption of serotonin inputs could dampen the strength of the LiCl. Implicit in this argument is that avoidance and aversion are on a continuum, as opposed to being qualitatively different processes, such that avoidance is rendered under less severe conditions than aversion. That is difficult to square with the fact that selective pharmacological disruption of insular cortex, for example, attenuate conditioned avoidance, without affecting conditioned aversive TR [40].

The present experiments attempted to tease these factors apart for GI-based stimuli utilizing a dose of LiCl that conditioned avoidance at a comparable rate to that of lactose. Here, we show that a dose of LiCl (0.3 mEq/kg) that, like lactose, conditions strong avoidance does not necessarily render the taste CS *aversive*. However, whereas lactose did not change ingestive TR, the intermediate dose of LiCl did significantly reduce ingestive responses. Another recent study assessed whether a GI stimulus that presumably produces a qualitatively distinct consequence (e.g., GI pain) from the emetic salt, LiCl, would render avoidance *with* or *without* aversion [36]. Rats conditioned to associate IO infusions of a saccharin CS with injections of hypertonic saline or LiCl gradually suppressed ingestive TR across trials; LiCl additionally fostered an increase in aversive TR that hypertonic saline did not [36]. Both stimuli led to complete or near avoidance of the CS in the first follow up intake test. Unfortunately, lack of intake (i.e., absence of a measurable response) does not provide a sensitive means for assessing the strength of a conditioned avoidance. While both the present results and those of Dwyer et al. [36] confirm that avoidance does not necessarily require a change in aversive reactivity, whether this is due to qualitative versus quantitative aspects of the GI stimuli used within and across these studies cannot be conclusively determined yet. What is clear from the individual data shown in each figure here (and summarized in S1 and S2 Figs) is that there is not always a clear correspondence between intake suppression and TR or PR across rats. For example, rats that show the most expedient avoidance do not necessarily display the most robust reductions in ingestive TR or increases in aversive TR. Whether the GI-based processes that underlie these appetitive versus consummatory responses are dissociable remains to be determined. Future studies will use alternative strategies to separate these stimulus features.

### Microstructural licking patterns versus taste reactivity

Some have argued that at least two features of licking behavior—the initial lick response (e.g., the first minute lick rate) and the overall burst size—are determined by the same taste properties that underlie the oromotor responses measured by the TR test [41–43]. This comparison is based on the fact that these microstructural components and ingestive TR increase with increasing concentrations of normally preferred tastants (i.e., sucrose) and decrease (while aversive TR increases) with increasing concentrations of normally avoided tastants (i.e., quinine). Moreover, when a normally preferred tastant (i.e., sucrose) is paired with LiCl, initial lick rate, lick burst size, ingestive TR all decrease, whereas burst number and aversive TR increase [5, 18, 44]. Experiment 2, therefore, explicitly compared the microstructural patterns of licking behavior on the final training trial across the three different training groups. These analyses showed that, as expected, initial lick rate and burst size were significantly reduced for the High LiCl group compared to the NaCl group. The two Lactose rats that showed the most dramatic reduction in CS intake not unexpectedly also exhibited a very low initial lick rate and took small bursts. Yet, even the Lactose rats that continued to consume some amount of saccharin on the last trial in Experiment 2 also had smaller initial lick rates and burst sizes than their NaCl counterparts. A recent study by Arthurs et al [27] also found that pairing 0.1% saccharin with, in their case, a higher dose of lactose via intragastric infusion reduced initial lick rate and burst size. Although these altered lick patterns were not directly compared to those associated with LiCl in that study, the authors concluded that the change in these two aspects of licking indicate that Lactose conditioned an *aversion* to the taste CS. Arthurs et al [27] argue that licking microstructure is sensitive to one dimension of decreased palatability but cannot detect changes in aversive reactivity. However, the fact we saw comparable reductions on burst size and initial lick rate in Experiment 2, but did not see any change in TR in Experiment 1, not even in terms of a reduction in ingestive TR, after lactose experience suggests these taste reactivity and microstructural patterns of licking are not always in register and may, in fact, be susceptible to different factors. Importantly, it is unclear whether burst size and number are systematically related to stimulus valence when intake is so low (as in conditioned avoidance). Considering both appetitive and consummatory processes affect licking patterns, the influence of each are not entirely dissociable in these licking microstructure measures. Taste reactivity, on the other hand, provides a readout of consummatory responses in the absence of an appetitive influence. Future studies will need to compare these two measures more directly, as differences may ultimately prove to have great interpretative value. Nevertheless, the present results caution against equating lick microstructure and taste reactivity patterns, especially in terms of their relationship to palatability.

### Contextual influences

One important factor to consider when comparing across these response measures (TR, intake, breakpoint) is context; that includes the exteroceptive context such as the room, chamber, fluid delivery system, and the interoceptive context, such as the deprivation state. Interoceptive and exteroceptive cues are encoded in learned associations and come to exert some control over responding [11, 12, 45–49]. Accordingly, one might predict that the shift, both exteroceptively and interoceptively, from the single-bottle training or testing to the TR test assay could effectively weaken the expression of conditioned rejection responses that were established in a different context, especially if the initial learning was less robust or otherwise different. The same case could be made for the post-training PR test, though in that situation, the interoceptive status remained consistent. Accordingly, one might speculate that the robust responses conditioned by High LiCl were simply less vulnerable to such shifts than the responses conditioned by Lactose. However, the Lactose group exhibited near complete avoidance of the CS in the final two-bottle choice test, which was also conducted in a distinct environment (ad lib and in the home cage). Thus, if context was the critical factor in gating the expression of the response, then that should reveal itself in the two-bottle test as well. The overall pattern of results in both experiments indicate that despite the fact that rats in both groups displayed near complete avoidance during intake and preference tests, the learning and/or expression of the learning was not identical between the High LiCl and Lactose conditions.

Deprivation state directly modulates ingestive motivation as well [26, 50–53]. Generally speaking, deprivation enhances appetitive and consummatory responses to some taste solutions, even some concentrations of normally avoided stimuli (e.g., hypertonic salt) [51, 53, 54]. Therefore, a lack of physiological need during the TR test would be expected to lower the threshold for rejection responses in all groups. Water-replete rats are in a better position to limit ingestive behaviors and increase rejection behavior for a taste CS associated with negative visceral consequences. Yet, even under these conditions, the Lactose rats exhibited high ingestive responding and no aversive responding.

### Effects on appetitive responding

Intake is an outcome measure that is typically cast as the product of consummatory and appetitive behavior. A factor that distinguishes aversion and avoidance is that only the former leads to concomitant changes in taste-guided oromotor reactivity in the consummatory domain of responding. By logical deduction, we, like others, reasoned that appetitive processes must, therefore, play a large role in the expression of a conditioned taste avoidance. Specifically, the theory posits that the visceral consequences of certain types of stimuli diminish the tendency to approach or otherwise obtain a stimulus that is known to produce the negative consequence, without necessarily diminishing the palatability of the CS.

There are a number of operant tasks used to measure such appetitive processes, whereby the performance of a specific response results in access to a stimulus (e.g., food, drug, usually called the reinforcer or outcome). The extent to and/or rate at which the response is performed varies as a function of stimulus, deprivation state, and task requirement (s) [55–58]. For example, operant responding increases as the concentration of a sucrose reinforcer increases and also is enhanced by food or water deprivation {e.g., [53, 59, 60]}. Conversely, operant responding decreases with increasing concentrations of a normally-avoided stimulus (e.g., quinine) or when the outcome reinforcer has been otherwise devalued [53, 60, 61]. Devaluation usually takes one of two forms. The food/fluid reinforcer is paired with LiCl or the food reinforcer is fed to the point of satiation ahead of the operant testing session. Both of these procedures significantly suppress subsequent behaviors geared at obtaining that reinforcer [62–64].

The PR breakpoint or the maximum amount of effort the subject is willing to expend in order to receive a particular outcome is a commonly used measure to assess the motivational potency of that outcome reinforcer (e.g., incentive value) [65]. Under certain conditions, incentive value is thought to be dissociable from hedonic value [66]. In line with this model, one hypothesis is that tastes associated with adverse consequences that do not seem to impact hedonic value, as inferred from taste reactivity measures, may still undergo a loss in incentive value or salience. Accordingly, we expected that both High LiCl and Lactose rats who learned to stop drinking the taste CS would show dramatic reductions in PR breakpoint. Although the sample size was small, the effect of taste-LiCl training on appetitive responding was evident in a significant reduction in breakpoint, as expected, as well as the tendency to rapidly reduce the volume and the rate at which the reinforcer (saccharin) was consumed (not statistically tested) (see S1 Fig).

Surprisingly, even the Lactose rats that strongly avoided drinking the taste CS during training and the single-bottle intake test showed no such decrement in breakpoint. Nor were other measures of performance in the PR task generally perturbed, relative to the NaCl group. The Lactose group generally took most, if not all, of the reinforcement licks; licks only started to decline as rats approached their breakpoint. The Lactose group also showed very rapid rates of responding as indicated by the interlick intervals both during the dry lick (operant) phase and during the reinforcement phase (see S3 Fig). Overall, the Lactose rats looked more like the NaCl control rats than the High LiCl rats on each of these measures. These “normal” behaviors were even observed in the two rats that showed the strongest avoidance in the intake training and test sessions. That said, two rats that had previously learned to reduce intake, but did not completely avoid the CS during the single-bottle conditioning and testing sessions, did appear to reduce their breakpoints (compared to the pre-conditioning baseline) after conditioning; thus, these rats resembled more of a “High LiCl profile” on the PR test. Nevertheless, the overall negative and variable results with the Lactose group suggest that conditioned taste avoidance (in intake tests) and PR are driven by dissociable factors.

To our knowledge, no study to date has explicitly tested whether other types of negative postoral consequences, like lactose malabsorption, are capable of devaluing food/fluid reinforcers in a way that influences behavior in other operant tasks. Nevertheless, there are some available data that speak to this issue. First, although moderate doses of LiCl are effective at devaluing a reinforcer, Ballenine and colleagues demonstrated that in order for that association to subsequently affect operant responding in an outcome devaluation test, the rat needs to have had re-experienced the reinforcer [67–69]. That is, whereas rats that have re-experienced the taste of the reinforcer after it was paired LiCl subsequently show dramatic reductions in operant responding on the manipulandum associated with that reinforcer, those that have not had the opportunity to re-experience the taste of the reinforcer continue to respond vigorously on the manipulandum associated with that outcome (in extinction). Accordingly, they have argued that contact with the reinforcer (e.g., a taste solution) allows the subject to experience its now *disgusting* taste properties, in effect updating its *hedonic* value, which, in turn, promotes the execution of the operant that leads to that outcome [67]. Moreover, treatment with Ondansetron, an anti-emetic agent, during the reinforcer—LiCl pairing phase attenuates the effect of the LiCl devaluation on subsequent operant responding [70]. That, coupled with the fact that this same type of antiemetic treatment given during taste aversion conditioning prevents the typical shift from taste CS-elicited ingestive TR to aversive TR, but does not interfere with CS avoidance in two-bottle choice tests [38], suggests a common underlying factor may influence the oromotor reflexes and the capacity of a stimulus to serve as a conditioned reinforcer.

### Variability in the efficacy of ID lactose to condition changes in intake

In both Experiments 1a and 2, we observed some degree of variability in terms of intake of saccharin paired with lactose. This could potentially reflect inherent individual differences in the ability to process lactose in the gastrointestinal tract; indeed, adult rats are known to have some residual lactase activity, and dietary and hormonal factors may influence the ability to tolerate lactose [17, 71–74]. Thus, it is possible that some rats were simply less adversely affected by the lactose dose infused. Alternatively, or perhaps additionally, there may be individual differences in the content of the learning. The training conditions used here were designed to mimic the typical CTA procedure, whereby the taste stimulus is consumed (via bottle) and the US (e.g., Lactose, LiCl, NaCl) is administered irrespective of how much the animal drinks (within limits, as described in the Materials and Methods). It is unclear whether the rats learn about the consequences of their appetitive and consummatory actions in these training sessions, but one can imagine a scenario in which the rats choose to suppress intake of the fluid that is associated with the negative consequences in order to avoid or at least minimize the insult. On the other hand, given the stimuli were delivered ID, another strategy would be to actually consume more of the CS, as that too would presumably dilute and therefore minimize the lactose load in the gut. In general, we did not see evidence of the latter (i.e., significant increases in intake with training); if anything, LiCl and Lactose attenuated intake.

### Summary and perspectives

The ability to immediately and proactively detect the presence of potentially harmful foods and limit contact and ingestion is extremely important for survival, yet compared to the study of beneficial foods and fluids, relatively little experimental attention has been paid towards understanding the associative and behavioral mechanisms underlying aversion and avoidance. Collectively, the behavioral dissociations shown here and in other studies underscore the need to consider these distinctions in studies aimed at clarifying the neural organization of taste-visceral integrative processes and highlight the need to incorporate these phenomena and other types of visceral signals more comprehensively into the current models of ingestive motivation.

## Supporting information

S1 Fig To permit comparison of behaviors for individual rats across the various outcome measures, each rat’s response is plotted for the major tests in Experiment 1a (left) and 1b (right). The order of rats from left to right across the x-axes are identical across panels. The last two digits of each rat’s ID number are given in the bottom graph. Histogram bars are color-coded by taste-ID conditioning group. Numerical digit 0 is displayed above the x axis when the outcome measure equaled zero.
(TIF)

S2 Fig To permit comparison of behaviors for individual rats across the various outcome measures, each rat’s response is plotted for the major tests in Experiment 2. The order of rats from left to right across the x-axes are identical across panels. The last two digits of each rat’s ID number are given in the bottom graph. Histogram bars are color-coded by taste-ID conditioning group. Numerical digit 0 is displayed above the x axis when the outcome measure equaled zero. The bottom panel shows the change in total number of reinforced lever presses (post-conditioning minus pre-conditioning). This was used to summarize the post-conditioning shift in breakpoint, but this metric was not used elsewhere in the paper.
(TIF)

S3 Fig Left column: Total number of reinforcements taken (in licks, out of a possible 15 licks) is plotted across each ratio trial completed for each individual rat in the High LiCl (red, **A**), Lactose (dark blue, **D**), and NaCl (medium blue, **G**) groups in Experiment 2. Middle Column: Each rat’s mean interlick interval (in milliseconds) on the reinforcement licks taken plotted as a function of ratio trial (High LiCl, **B**; Lactose, **E**; NaCl, **H**). Right column: Each rat’s mean interlick interval (in milliseconds) on the dry (operant) licks required on each ratio trial (High LiCl, **C**; Lactose, **F**; NaCl, **I**). Note that the symbol for a given rat within each group corresponds across graphs in this figure and other figures.
(TIF)
