# Supplementary figures and images for: Conditioned taste aversion versus avoidance: A re-examination of the separate processes hypothesis
Source: PLoS One. 2019 Jun 19;14(6):e0217458. doi: 10.1371/journal.pone.0217458 (PMC6583984; doi:10.1371/journal.pone.0217458)

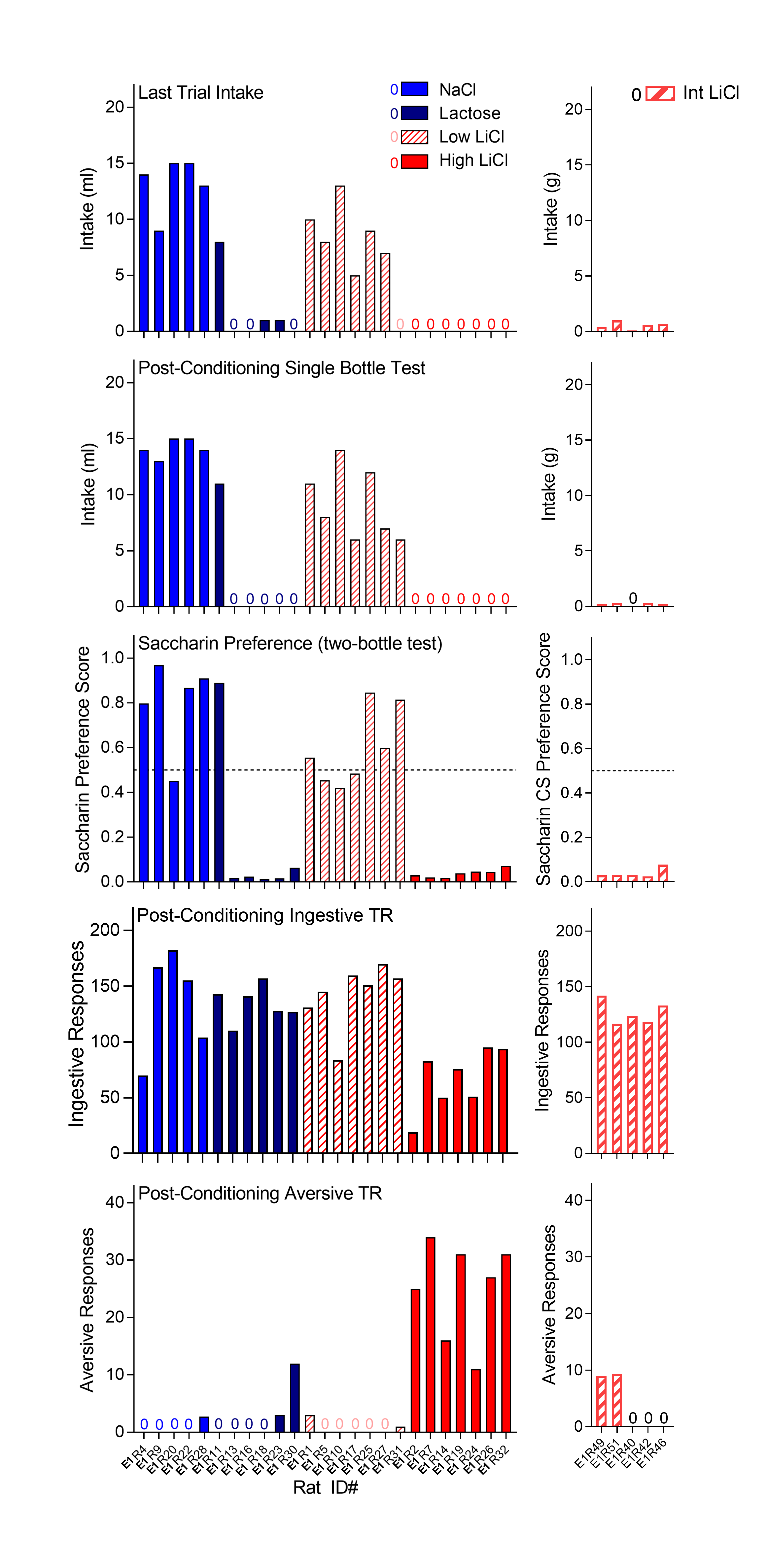

Supplement: S1 Fig — To permit comparison of behaviors for individual rats across the various outcome measures, each rat’s response is plotted for the major tests in Experiment 1a (left) and 1b (right). The order of rats from left to right across the x-axes are identical across panels. The last two digits of each rat’s ID number are given in the bottom graph. Histogram bars are color-coded by taste-ID conditioning group. Numerical digit 0 is displayed above the x axis when the outcome measure equaled zero. (TIF) [file pone.0217458.s001.tif]

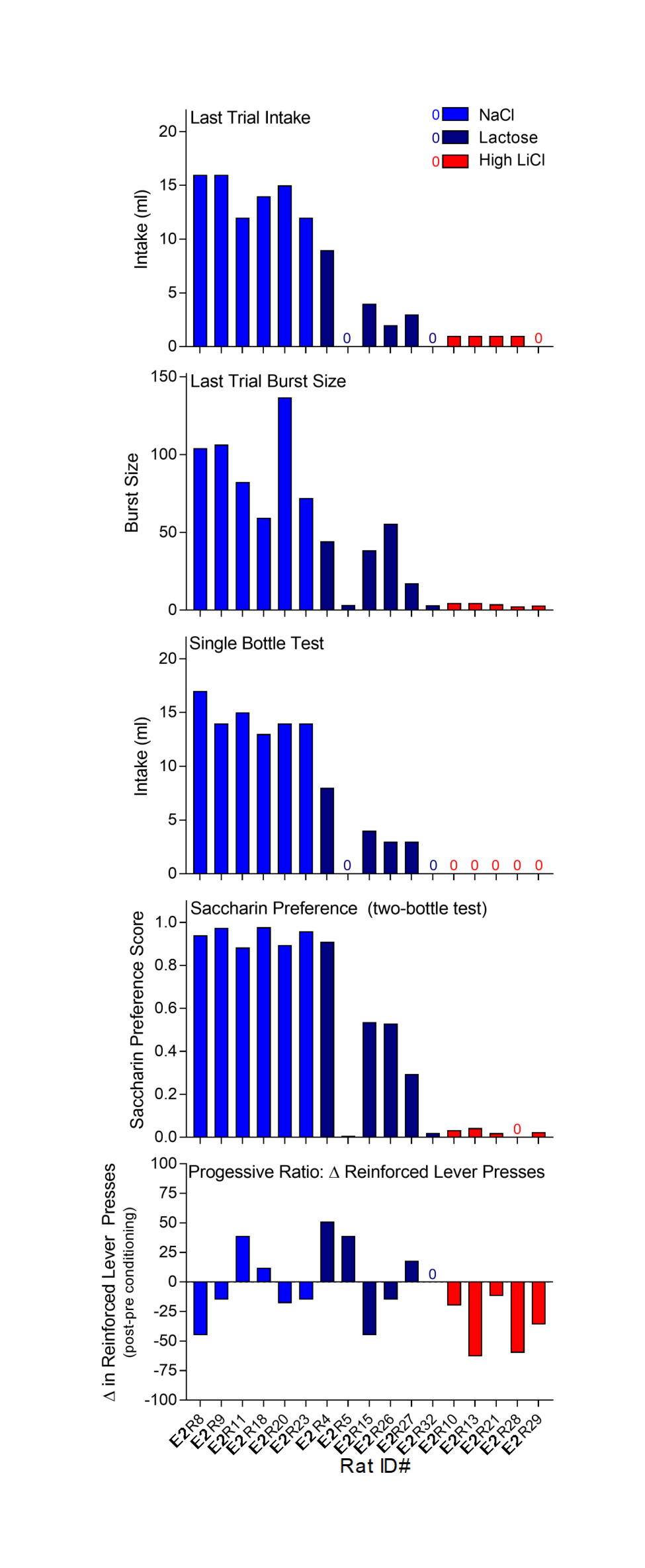

Supplement: S2 Fig — The order of rats from left to right across the x-axes are identical across panels. The last two digits of each rat’s ID number are given in the bottom graph. Histogram bars are color-coded by taste-ID conditioning group. Numerical digit 0 is displayed above the x axis when the outcome measure equaled zero. The bottom panel shows the change in total number of reinforced lever presses (post-conditioning minus pre-conditioning). This was used to summarize the post-conditioning shift in breakpoint, but this metric was not used elsewhere in the paper. (TIF) [file pone.0217458.s002.tif]

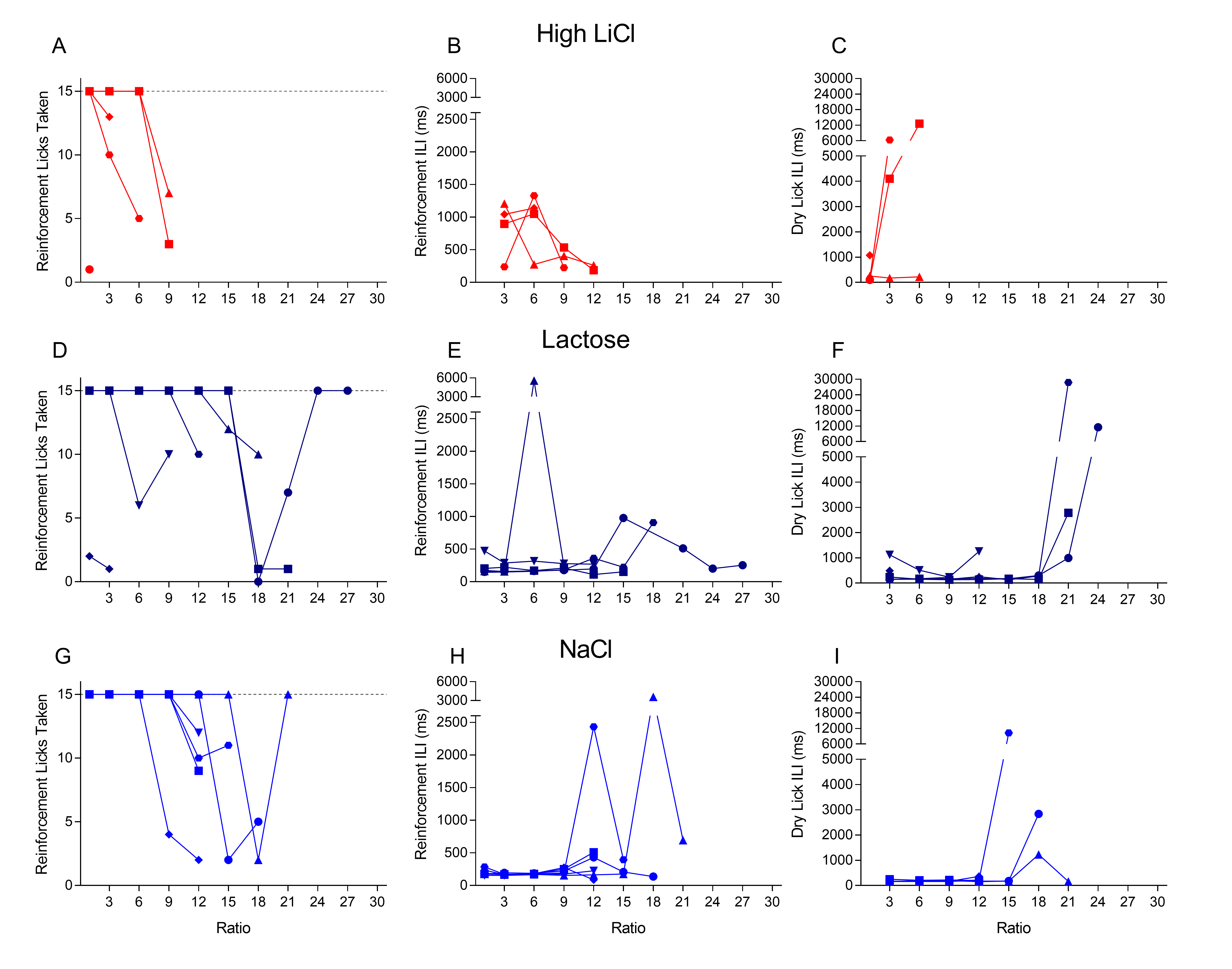

Supplement: S3 Fig — Left column: Total number of reinforcements taken (in licks, out of a possible 15 licks) is plotted across each ratio trial completed for each individual rat in the High LiCl (red, A), Lactose (dark blue, D), and NaCl (medium blue, G) groups in Experiment 2. Middle Column: Each rat’s mean interlick interval (in milliseconds) on the reinforcement licks taken plotted as a function of ratio trial (High LiCl, B; Lactose, E; NaCl, H). Right column: Each rat’s mean interlick interval (in milliseconds) on the dry (operant) licks required on each ratio trial (High LiCl, C; Lactose, F; NaCl, I). Note that the symbol for a given rat within each group corresponds across graphs in this figure and other figures. (TIF) [file pone.0217458.s003.tif]
